# Lactose-reduced infant formula with added corn syrup solids is associated with a distinct gut microbiota in Hispanic infants

**DOI:** 10.1080/19490976.2020.1813534

**Published:** 2020-09-04

**Authors:** Roshonda B. Jones, Paige K. Berger, Jasmine F. Plows, Tanya L. Alderete, Joshua Millstein, Jennifer Fogel, Stanislav N. Iablokov, Dmitry A. Rodionov, Andrei L. Osterman, Lars Bode, Michael I. Goran

**Affiliations:** a Department of Pediatrics, The Saban Research Institute, Children’s Hospital Los Angeles, University of Southern California, Los Angeles, CA, USA; b Department of Integrative Physiology, University of Colorado Boulder, Boulder, CO, USA; c Department of Preventive Medicine, University of Southern California, Los Angeles, CA, USA; d A. A. Kharkevich Institute for Information Transmission Problems, Russian Academy of Sciences, Moscow, Russia; e P.G. Demidov Yaroslavl State University, Yaroslavl, Russia; f Sanford Burnham Prebys Medical Discovery Institute, La Jolla, CA, USA; g Department of Pediatrics and Larsson-Rosenquist Foundation Mother-Milk-Infant Center of Research Excellence, University of California, San Diego, CA, USA

**Keywords:** Infant, infant formula, human milk, sugar, Hispanic

## Abstract

Infant formula feeding, compared with human milk, has been associated with development of a distinct infant gut microbiome, but no previous study has examined effects of formula with added sugars. This work examined differences in gut microbiota among 91 Hispanic infants who consumed human milk [at breast (BB) vs. pumped in bottle (BP)] and 2 kinds of infant formula [(traditional lactose-based (TF) vs. lactose-reduced with added sugar (ASF)]. At 1 and 6 months, infant stool was collected to characterize gut microbiota. At 6 months, mothers completed 24-hour dietary recalls and questionnaires to determine infant consumption of human milk (BB vs. BP) or formula (TF vs. ASF). Linear regression models were used to determine associations of milk consumption type and microbial features at 6 months. Infants in the formula groups exhibited a significantly more ‘mature’ microbiome than infants in the human milk groups with the most pronounced differences observed between the ASF vs. BB groups. In the ASF group, we observed reduced log-normalized abundance of *Bifidobacteriaceae* (TF-BB Mean Difference = −0.71, ASF-BB Mean Difference = −1.10), and increased abundance of *Lachnospiraceae* (TF-BB Mean Difference = +0.89, ASF-BB Mean Difference = +1.20). We also observed a higher Community Phenotype Index of propionate, most likely produced by *Lachnospiraceae*, in the ASF group (TF-BB Mean Difference = +0.27, ASF-BB Mean Difference = +0.36). This study provides the first evidence that consumption of infant formula with added sugar may have a stronger association than birth delivery mode, infant caloric intake, and maternal BMI on the infant’s microbiome at 6 months of age.

## Introduction

Factors that impact the composition of the infant gut microbiome include delivery mode,^1^ gestational age,^2,3^ genetics,^4^ antibiotic use, maternal diet,^5,6^ and infant feeding type (e.g., human milk vs. formula).^7,8^ While more recent studies suggest that infant feeding practices may be the largest driver of gut microbial development, earlier studies have compared human milk feeding versus formula feeding in general without considering the effects of infant formulas that contain added sugars.

Despite well-known differences in the gut microbiota between human milk-fed and formula-fed infants, the impact of early introduction of added sugars in formula on the development of the infant gut microbiota has not been previously examined. Formulas with added sugar are labeled as “gentle” and marketed to improve colic or “fussiness” in infants by removal of lactose.^9,10^ However, these “gentle” formulas are distinct from other traditional formulas because they contain lower concentrations of lactose (and therefore galactose as well), often displaced by added sugar in the form of corn syrup solids. Infants who are exposed to lactose-reduced formula with added sugar can conceivably have an altered gut microbiome, as has been revealed in animal studies that examined the microbiome of juvenile rodents.^11^ To our knowledge, no human studies have examined the influence of lactose-reduced formula with added sugar on gut microbiota of infants. Therefore, the primary aim of this work was to examine differences in gut microbiota of infants that consumed: (1) human milk directly from breast and no formula; (2) pumped human milk delivered to the infant from a bottle and no formula; (3) traditional lactose-based formula; and (4) lactose-reduced formula with added sugar.

## Results

### General characteristics of infant cohort

This study included data from 91 Hispanic infants from the Los Angeles area. Infants were grouped by milk consumption type at 6 months of age: those who consumed human milk directly from breast and no formula (BB, n = 14), those who consumed pumped human milk and no formula (BP; n = 19), those who consumed a traditional formula (TF; n = 30) and those who consumed a lactose-reduced formula with added corn syrup solids formula (ASF; n = 29). Information regarding macronutrient composition and average macronutrient intake of infants by feeding type are provided in Supplemental Table 1. Briefly, the lactose-reduced formula with added sugar contains more glucose and maltose, has a higher glycemic index, and contains more added sugar (as measured by available carbohydrates) vs. traditional lactose-based formula and human milk. Table 1 provides a summary of participants’ demographics, anthropometrics, body composition at 6 months of age by feeding type. Statistically significant differences between feeding-type groups were observed for mother’s BMI so this was included as a covariate in the analysis (Table 1).

**Table 1.** General characteristics of 6-month old infants by milk consumption.

	BB (n = 14)	BP (n = 19)	TF (n = 30)	ASF (n = 28)	*P*-value ^A^
***General Characteristics***					
Sex (F/M)^B^	6/8	12/7	12/18	17/11	0.26
Age (days)	***181 (5.6)***	***183 (5.7)***	***188 (9.9)***	***185 (8.3)***	***0.04***
***Body Composition***					
Weight (kg)	7.8 (0.78)	8 (0.72)	8.1 (0.61)	7.9 (0.84)	0.6
Length (cm)	67 (2.4)	67 (2.2)	67 (1.6)	66 (2.2)	0.90
BMI z-score	0.21 (1.4)	0.65 (0.87)	0.68 (0.89)	0.59 (0.93)	0.52
Weight-for-length z-score	0.3 (1.5)	0.74 (0.87)	0.76 (0.88)	0.69 (0.91)	0.53
Weight z-score	0.18 (0.97)	0.53 (0.7)	0.39 (0.64)	0.39 (0.74)	0.61
Length z-score	0.039 (1.2)	0.084 (0.93)	−0.21 (0.7)	−0.11 (0.99)	0.71
Tricep Skinfold (mm)	8.5 (1.3)	8.3 (2.4)	9 (2.6)	9.1 (2.4)	0.62
Subscapular Skinfold (mm)	7.9 (1.8)	7.9 (2.7)	7.9 (2)	8 (2)	1.00
Suprailiac Skinfold (mm)	6 (2)	6.3 (2.3)	6.2 (2.7)	5.9 (1.5)	0.93
Midthigh Skinfold (mm)	19 (3.8)	21 (3.2)	20 (3.7)	21 (4.1)	0.36
Abdominal Circumference (cm)	41 (2.4)	42 (2.6)	42 (3.1)	41 (2.7)	0.36
***Infant Rate of Growth from Birth***					
**Difference in weight-for-age z-score**	−0.17 (0.97)	0.64 (0.76)	0.087 (0.98)	0.19 (1)	0.09
**Difference in BMI-for-age z-score**	0.38 (1.7)	1.1 (1.1)	0.5 (1.2)	0.65 (1.2)	0.38
**Difference in weight-for-length z-score**	0.82 (1.7)	1.3 (1.5)	0.71 (1.5)	0.86 (1.3)	0.60
***Maternal Factors***					
Age (years)	30 (6)	29 (6.2)	28 (5.5)	31 (6.6)	0.32
BMI	***26 (5.4)***	***30 (4.9)***	***32 (5.4)***	***31 (5.3)***	***0.006***
Pre-pregnancy BMI	***24 (5)***	***28 (5.3)***	***30 (5.3)***	***28 (5.7)***	***0.03***
Mode of Delivery (Vaginal/C-section)^B^	11/3	18/1	23/7	19/9	0.19
***Macronutrient Intake***					
Solid Introduction (Yes/No)^B^	9/5	16/3	27/3	24/4	0.19
Total Caloric Intake (kCal)	689 (166)	680 (180)	673 (111)	667 (139)	0.97

^A^*P*-values from an ANOVA testing for differences in means by milk consumption type (or from Chi-square test for differences in frequency by milk consumption type if variable is categorical).

Data is expressed as Mean (SD) or ^B^frequency by milk consumption type if variable is categorical.

BB = Human milk directly from breast only (No formula)

BP = Human milk from a bottle (No formula)

TF = Traditional formula

ASF = Lactose-reduced formula with added corn syrup solids

### Differences in diversity of gut microbiota at 6 months of infant age

**Figure 1.**
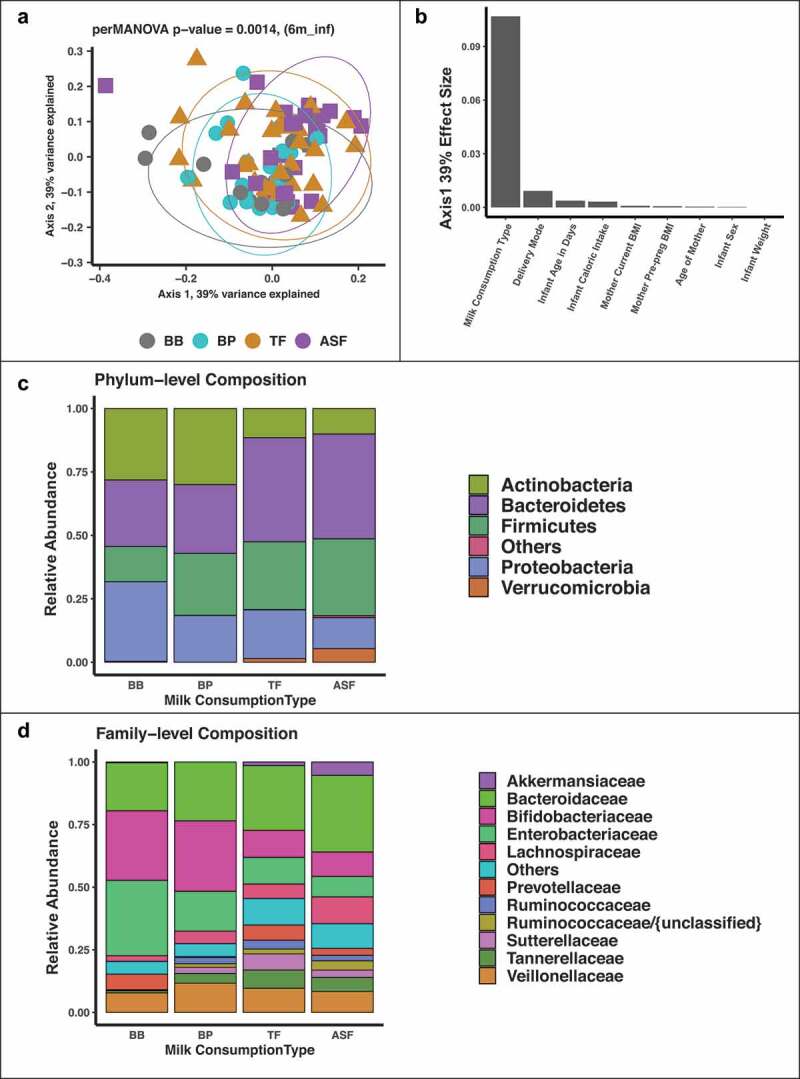
Multidimensional scaling (MDS) of 16S rRNA gene sequences colored by milk consumption type at 6 months. There is statistically significant separation between formula-fed infants and breastfed infants for both MDS axis 1 and axis 2, *p* values computed by independent ANOVA tests of each axis (a). Milk consumption type has a larger effect on the first MDS axis than delivery mode and infant weight (b). The phylum Proteobacteria has the highest mean relative abundance in the breast-fed (BB) group while Bacteroidetes has the highest mean relative abundance in the two formula groups (TF and ASF) (c). The family *Enterobacteriaceae* has the highest mean relative abundance in the BB group while *Bacteroidaceae* has the highest mean relative abundance in TF and ASF groups (d).

At 6 months of age, there was no statistically significant difference in alpha diversity between milk consumption type as measured by Shannon’s index, Simpson’s index, richness, evenness, and total sequence reads per samples (Table 2). To examine the beta diversity of the infants’ microbiome, we applied multidimensional scaling of the ASVs using DEICODE ^12^ and examined the first three axes which explained ~100% of the variation. At 6 months of infant age, there was a significant difference in the beta diversity in the gut microbiota between human milk and formula (R^2^ = 0.11, *p* value = 0.002) but not between the two formula types (R^2^ = 0.033, *p* value = 0.1518) or between the two modes of breast delivery (R^2^ = 0.00378, *p* value = 0.92). Milk consumption type had a larger effect (Beta = 0.11, *p* value = 0.02) on the composition of the microbiome than birth delivery mode, maternal BMI and infant caloric intake (Figure 1).

**Table 2.** Average diversity across milk consumption type.

	BB (n = 14) Mean (SD)	BP (n = 20) Mean (SD)	TF (n = 30) Mean (SD)	ASF (n = 27) Mean (SD)	*P*-value	Pairwise Group Differences Mean Difference					
						BP-BB	BB-TF	BP-TF	ASF-BB	ASF-BP	ASF-TF
***Alpha diversity***											
Shannon Index	1.7 (0.34)	2 (0.52)	2.3 (0.44)	2.4 (0.49)	0.231	0.30****	0.54****	0.24****	0.71****	0.41****	0.17****
Richness	29 (10)	37 (16)	44 (11)	52 (26)	0.292	7.7****	15****	7.1****	23****	15****	7.9****
Evenness	0.51 (0.074)	0.56 (0.11)	0.6 (0.098)	0.62 (0.099)	0.246	0.05****	0.088****	0.038****	0.11****	0.062****	0.024****
Sequence reads	26278 (2344)	27025 (3054)	25480 (4176)	25094 (3863)	0.246	747***	−787****	−1534****	−1184****	−1931****	−397*
***Beta diversity***											
Axis 1 (39% ^A^)	−0.043 (0.12)	−0.029 (0.071)	9.0 x 10^−4^ (0.1)	0.043 (0.11)	0.0143	0.021	0.05	0.029	0.096	0.075	0.046
Axis 2 (39% ^A^)	−0.042 (0.084)	−0.034 (0.1)	0.011 (0.1)	0.035 (0.11)	0.0896	0.035	0.079*	0.044	0.089*	0.054	0.01
Axis 3 (22% ^A^)	−0.036 (0.079)	−0.032 (0.091)	0.0065 (0.11)	0.036 (0.12)	0.102	0.0035	0.042	0.038	0.075	0.071	0.033

^A^Percent of variation explained

* = *P* < 0.05; ** = *P* < 0.01; *** = *P* < 0.001; **** = *P* < 0.0001

BB = Human milk directly from breast only (No formula)

BP = Human milk from a bottle (No formula)

TF = Traditional Formula

ASF = Lactose-reduced formula with added corn syrup solids

SD = Standard Deviation

### Differences in abundances of gut microbiota at 6 months of infant age

**Figure 2.**
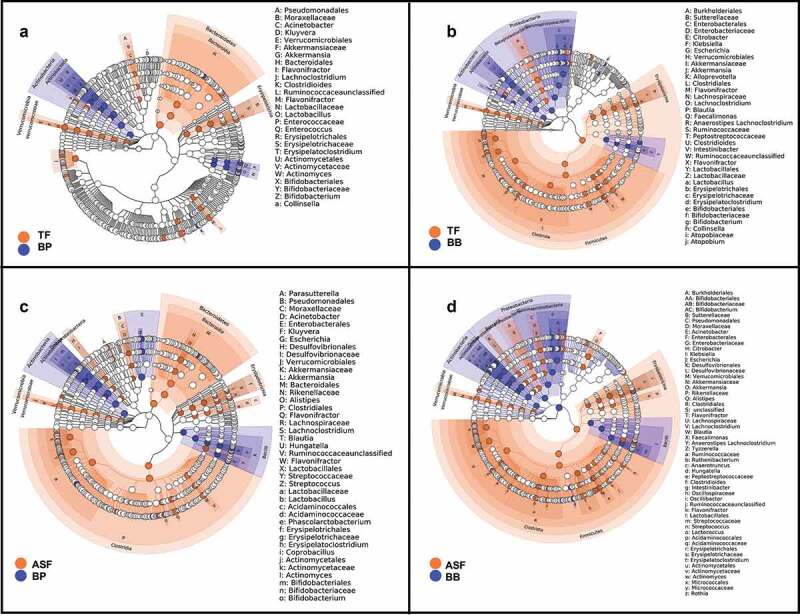
Linear discriminant analysis shows the microbial taxonomic groups that are enriched and depleted in infants consuming formula versus infants whose sole milk source is human milk. When comparing the gut microbiome of infants in the who consume expressed human milk in a bottle (BP) to infants who consume traditional formula (TF) (a), differences are apparent, but more differences result from the comparison of the gut microbiomes of infants whose only source of milk is through breastfeeding (BB) to those in the TF group (b). Even more microbes are revealed to be discriminant when we compare BP group and group consuming lactose-reduced formula with added-sugar (ASF) (c) and the BB group and ASF (d).

**Figure 3.**
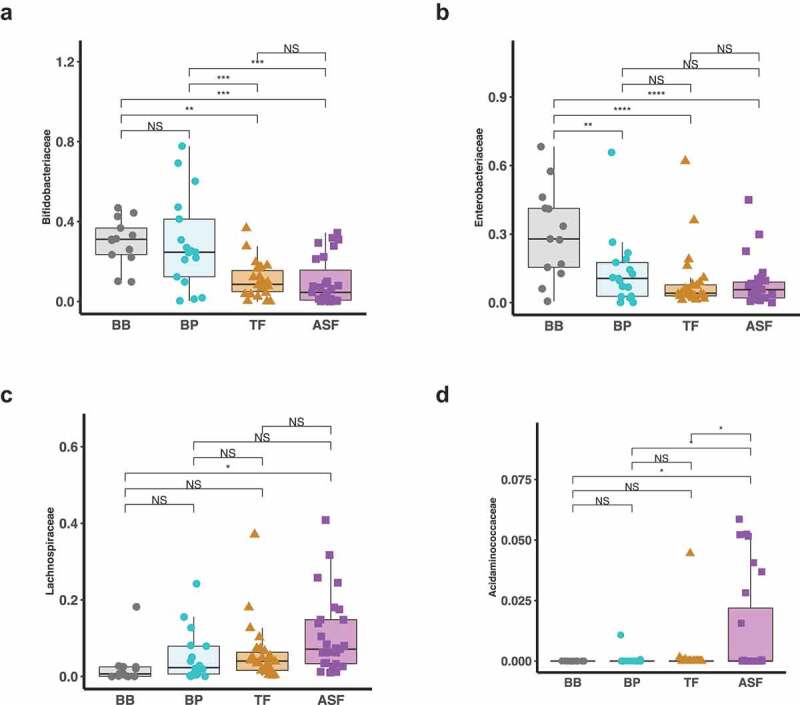
Log-normalized relative abundances of taxonomic families that are significantly different among the milk consumption types. At 6 months, microbes belonging to *Bifidobacteriaceae* are enriched in breastfed infants compared to formula fed infants with the infants who consume lactose-reduced formula with added corn syrup solids (ASF) having the lowest abundance (a). *Enterobacteriaceae* was also enriched in breastfed infants compared to formula fed infants but it was also significantly lower in the group who consume some human milk from a bottle (b). Further, microbes belonging to the families *Lachnospiraceae* are enriched in formula fed infants compared to breastfed infants with the infants in the ASF group having the highest abundance (c). *Acidaminococcaceae* was significantly higher in the infants who consume lactose-reduced formula with added corn syrup solids (ASF) (d).

We identified microbes that were differentially enriched or depleted in the four infant feeding groups. Using LEfSe discriminant analysis, we observed taxonomic groups in which pairwise differences in the milk consumption groups were discriminant among the groups (Figure 2). We further examined the differences in abundances of each microbe using linear regression models adjusting for covariates (Supplemental Table 2–5). In particular, we found that relative to human milk (BB and BP), infants who consumed formula (TF and ASF) had a lower relative abundance of the family *Bifidobacteriaceae* (TF-BB Mean Difference = −0.71, HSD *p* value > 0.05; ASF-BB Mean Difference = −1.10, HSD *p* value = 0.002; TF-BP Mean Difference = −0.49, HSD *p* value > 0.05; ASF-BP Mean Difference = −0.83, HSD *p* value = 0.01). The depletion of *Bifidobacteriaceae* relative to the human milk groups was greater in the ASF than the depletion when compared to the TF group. Also, we found that formula-fed infants had significantly increased abundances of ASVs assigned to the family *Lachnospiraceae* (TF-BB Mean Difference = +0.89, HSD *p* value = 0.001; ASF-BB Mean Difference = +1.20, HSD *p* value = 3.3 × 10^−6^; TF-BP Mean Difference = −0.23, HSD *p* value > 0.05, ASF-BP Mean Difference = +0.58, HSD *p* value = 0.03). This relative enrichment was greater in ASF than TF. We also found that when compared to the group consuming traditional formula (TF), there was a significant increase in the family *Acidaminococcaceae* in the group of infants consuming lactose-reduced formula with added corn syrup solids (ASF-TF Difference = +0.71, HSD *p* value = 0.03) (Table 3, Figure 3). These findings were independent of infant’s sex, age in days, infant weight and abundance of the respective gut microbe at 1 month and also independent of mother’s age, current BMI, prepregnancy BMI, and delivery mode.

**Table 3.** Taxonomic families determined to be significantly different among milk consumption type groups.

*Taxonomic family*	BB (n = 14) Mean (SD)	BP (n = 20) Mean (SD)	TF (n = 30) Mean (SD)	ASF (n = 27) Mean (SD)	*P*-value	Pairwise Group Differences Mean Difference					
						BP-BB	BB-TF	BP-TF	ASF-BB	ASF-BP	ASF-TF
*Ruminococcaceae{unclassified}*	0.23 (0.61)	0.77 (1.2)	2.1 (1.1)	2.4 (1)	2.8 x 10^−8^	0.82	2.2****	1.3***	2.3****	1.5****	0.17
*Lachnospiraceae*	2 (1)	2.5 (1)	2.9 (0.6)	3.2 (0.45)	2.5 x 10^−5^	0.66*	0.89**	0.23	1.2****	0.58*	0.34
*Peptostreptococcaceae*	0.56 (0.69)	0.91 (1)	1.3 (0.64)	1.6 (0.51)	1.7 x 10^−4^	0.15	0.72*	0.57*	0.95***	0.8**	0.23
*Enterobacteriaceae*	3.7 (0.53)	3.3 (0.74)	3.2 (0.44)	3 (0.65)	2.6 x 10^−3^	−0.52*	−0.59**	−0.07	−0.71***	−0.19	−0.12
*Bifidobacteriaceae*	3.7 (0.63)	3.6 (0.6)	3.2 (0.81)	2.8 (1.1)	2.9 x 10^−3^	−0.22	−0.71	−0.49	−1.1**	−0.83*	−0.34
*Lactobacillaceae*	0.99 (0.8)	1.4 (0.83)	0.45 (0.65)	0.6 (0.7)	3.5 x 10^−3^	0.33	−0.65*	−0.99***	−0.47	−0.8**	0.19
*Acidaminococcaceae*	0.17 (0.64)	0.19 (0.61)	0.23 (0.67)	0.97 (1.4)	5.0 x 10^−3^	0.22	0.26	0.04	0.97*	0.75*	0.71*
*Erysipelotrichaceae*	0.62 (1)	0.63 (0.91)	1.3 (0.9)	1.6 (0.7)	7.3 x 10^−3^	0.074	0.81*	0.74*	0.95*	0.88*	0.14
*Micrococcaceae*	0.66 (0.72)	0.34 (0.62)	0.27 (0.58)	0.12 (0.36)	0.015	−0.39	−0.57**	−0.19	−0.59**	−0.2	−0.012
*Atopobiaceae*	0.67 (0.94)	0.3 (0.74)	0.16 (0.5)	0.25 (0.63)	0.028	−0.61*	−0.62*	−0.00085	−0.47	0.14	0.15
*Coriobacteriaceae*	0.55 (0.93)	0.57 (0.91)	1.3 (1)	0.81 (0.91)	0.036	−0.041	0.79	0.83*	0.22	0.26	−0.57

* = *P* < 0.05; ** = *P* < 0.01; *** = *P* < 0.001; **** = *P* < 0.0001

BB = Human milk directly from breast only (No formula)

BP = Human milk from a bottle (No formula)

TF = Traditional Formula

ASF = Lactose-reduced formula with added corn syrup solids

SD = Standard Deviation

### Differences in predicted metabolic functions and phenotypes at 6 months

Finally, to examine differences in the functional potential of gut bacteria by milk consumption type, we applied the phenotype profiling approach using the obtained taxa relative abundances to quantify the fractional representation of predicted metabolic features (phenotypes) in the analyzed microbiome samples. In this analysis, we extended the approach previously described for B vitamins^13,14^ toward a tentative prediction of requirements and utilization capabilities for major nutrients (amino acid and carbohydrates) as well as a potential to produce major short chain fatty acids (SCFAs). The details of our computational approach, which is based on in silico pathway reconstruction^15-17^ and is fundamentally similar to a broadly utilized analysis of pathway abundance (as implemented via combination of PICRUSt and MinPath^18,19^ (are outlined in Methods, and the results are summarized in Supplemental Table 6. We have identified significant differences among the distinct milk consumption groups in predicted metabolic capabilities of their respective microbial communities pertaining to a subset of 25 metabolites spanning: (i) utilization of 19 mono- and oligosaccharides (ii) biosynthesis of cysteine, threonine and B12; (iii) lysine and methionine degradation; and (iv) propionate production (Table 4).

**Table 4.** Predicted community metabolic phenotypes determined to be significantly different among milk consumption type groups.

Community Phenotype Indices (CPIs)	BB (n = 14) Mean (SD)	BP (n = 20) Mean (SD)	TF (n = 30) Mean (SD)	ASF (n = 27) Mean (SD)	*P*-value	Pairwise Group Differences Mean Difference					
						BP-BB	BB-TF	BP-TF	ASF-BB	ASF-BP	ASF-TF
**Carbohydrate utilization**											
Galactitol	0.26 (0.15)	0.15 (0.17)	0.085 (0.12)	0.056 (0.044)	3.4 x 10^−6^	−0.13**	−0.19****	−0.057	−0.2****	−0.071	−0.013
Glucoronides	0.25 (0.17)	0.14 (0.17)	0.069 (0.11)	0.039 (0.03)	4.7 x 10^−6^	−0.12**	−0.19****	−0.071	−0.21****	−0.087*	−0.017
Lactose	0.61 (0.27)	0.5 (0.24)	0.28 (0.16)	0.26 (0.18)	8.7 x 10^−6^	−0.14	−0.37****	−0.23**	−0.37****	−0.23**	0.0015
Glucosamine	0.27 (0.16)	0.17 (0.17)	0.1 (0.13)	0.066 (0.065)	9.9 x 10^−5^	−0.12*	−0.18****	−0.056	−0.21****	−0.084	−0.027
Mannitol	0.37 (0.17)	0.26 (0.18)	0.19 (0.15)	0.16 (0.1)	1.3 x 10^−4^	−0.14	−0.21***	−0.067	−0.22***	−0.079	−0.012
N-acetylgalactosamine	0.24 (0.14)	0.14 (0.15)	0.089 (0.11)	0.056 (0.06)	1.4 x 10^−4^	−0.11*	−0.16***	−0.051	−0.18****	−0.074	−0.023
Psicoselysine	0.17 (0.12)	0.11 (0.11)	0.06 (0.08)	0.038 (0.03)	3.6 x 10^−4^	−0.075*	−0.12****	−0.043	−0.13****	−0.057	−0.015
Alpha-xylosides	0.31 (0.21)	0.16 (0.16)	0.14 (0.18)	0.086 (0.12)	4.4 x 10^−4^	−0.17*	−0.17*	0.0024	−0.23***	−0.056	−0.059
Gluconate	0.37 (0.2)	0.29 (0.21)	0.18 (0.16)	0.15 (0.13)	1.6 x 10^−3^	−0.094	−0.21***	−0.12	−0.23***	−0.13*	−0.013
Trehalose	0.33 (0.2)	0.19 (0.2)	0.16 (0.2)	0.12 (0.11)	2.4 x 10^−3^	−0.16*	−0.18**	−0.019	−0.21**	−0.046	−0.028
Fructoselysine	0.21 (0.12)	0.13 (0.12)	0.12 (0.12)	0.089 (0.06)	3.0 x 10^−3^	−0.096*	−0.11*	−0.01	−0.13**	−0.03	−0.02
Raffinose	0.2 (0.13)	0.23 (0.21)	0.088 (0.08)	0.087 (0.11)	3.1 x 10^−3^	0.028	−0.12	−0.14**	−0.13*	−0.16**	−0.012
Melibiose	0.25 (0.17)	0.15 (0.14)	0.12 (0.14)	0.1 (0.11)	3.8 x 10^−3^	−0.12	−0.14	−0.024	−0.15**	−0.03	−0.0063
Beta-arabinosides	0.14 (0.097)	0.15 (0.15)	0.052 (0.057)	0.05 (0.08)	8.1 x 10^−3^	0.003	−0.088	−0.091*	−0.097*	−0.1**	−0.0084
Sorbitol	0.31 (0.19)	0.2 (0.19)	0.15 (0.15)	0.18 (0.13)	9.9 x 10^−3^	−0.13	−0.17**	−0.04	−0.13*	−0.0025	0.038
Arabinose	0.69 (0.16)	0.51 (0.22)	0.45 (0.22)	0.5 (0.18)	0.016	−0.16	−0.21*	−0.047	−0.18*	−0.014	0.034
Fructose	0.94 (0.032)	0.87 (0.14)	0.82 (0.16)	0.78 (0.15)	0.017	−0.095	−0.12	−0.024	−0.17**	−0.071	−0.048
Lacto-N-biose	0.28 (0.16)	0.28 (0.18)	0.17 (0.13)	0.17 (0.14)	0.026	−0.015	−0.14	−0.12	−0.13	−0.12	0.0021
Rhamnose	0.52 (0.19)	0.37 (0.22)	0.43 (0.21)	0.48 (0.21)	0.047	−0.12	−0.05	0.068	−0.015	0.1	0.035
**SCFA utilization**											
Propionate	0.37 (0.22)	0.5 (0.24)	0.58 (0.23)	0.7 (0.19)	5.9 x 10^−5^	0.15	0.27***	0.12	0.36****	0.21**	0.089
**Amino Acid synthesis**											
Cysteine	0.65 (0.19)	0.61 (0.25)	0.79 (0.15)	0.8 (0.15)	2.1 x 10^−3^	−0.033	0.18*	0.22**	0.17*	0.21**	−0.0078
Threonine	0.99 (0.014)	0.99 (0.021)	0.97 (0.072)	0.93 (0.14)	6.0 x 10^−3^	−0.0054	−0.02	−0.014	−0.068*	−0.062*	−0.048*
**Amino Acid degradation**											
Lysine	0.3 (0.19)	0.18 (0.18)	0.13 (0.15)	0.13 (0.11)	1.9 x 10^−3^	−0.15*	−0.19***	−0.039	−0.17**	−0.026	0.013
Methionine	0.3 (0.18)	0.29 (0.24)	0.12 (0.11)	0.12 (0.14)	2.3 x 10^−3^	−0.017	−0.2**	−0.18**	−0.19**	−0.18**	0.007
**Vitamin synthesis**											
B12 (cobalamin)	0.3 (0.21)	0.45 (0.23)	0.53 (0.21)	0.65 (0.17)	4.9 x 10^−5^	0.17	0.29***	0.11	0.38****	0.2**	0.92

* = *P* < 0.05; ** = *P* < 0.01; *** = *P* < 0.001; **** = *P* < 0.0001

BB = Human milk directly from breast only (No formula)

BP = Human milk from a bottle (No formula)

TF = Traditional Formula

ASF = Lactose-reduced formula with added corn syrup solids

SD = Standard Deviation

**Figure 4.**
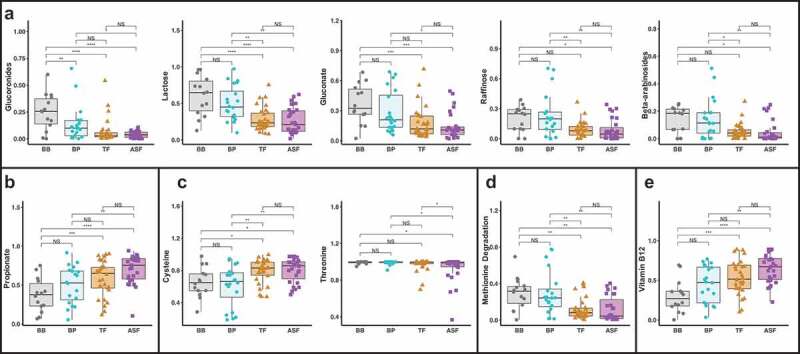
CPIs of predicted functions that are significantly different among the four milk consumption types. Gut microbial profiles derived from 16S rRNA sequencing of stool samples from 6 month old infants were used to predict functional phenotypes of the gut microbes. There were significant differences in carbohydrate utilization (a), short-chain fatty acid (SCFA) production (b), amino acid biosynthesis and degradation (C and D) and vitamin biosynthesis (e).

Among such discriminating metabolic capabilities quantified by Community Phenotype Indices (CPI, on the scale from 0 to 100% fractional representation) is lactose utilization showing a two-fold elevated CPI value in breastfed groups (BB and BP) compared to formula-fed groups (TF and ASF). The predicted utilization capabilities for glucuronides, gluconate, beta-arabinosides, and raffinose, were also elevated two- to five-fold in both human milk fed groups. CPI for methionine degradation was 2.5-fold higher in both human milk fed groups, whereas the biosynthetic propensity for cysteine and cobalamin (vitamin B12) biosynthesis were decreased 1.5- to 2-fold in the human milk fed groups. Predicted propionate production capability appeared to be ~1.5- to 2-fold higher in the formula-fed groups, but was also elevated in babies who consumed pumped human milk from a bottle (Figure 4).

## Discussion

This study provides the first evidence that infant formula with added sugar compared to traditional formula and human milk may have a stronger association than birth delivery mode, infant caloric, and maternal BMI on the infant’s microbiome. Differences in the infant microbiome were observed between the various feeding groups examined in this study, with formula-fed infants exhibiting a distinct microbiome with characteristics of premature maturation that is even more pronounced in infants fed formula with added sugars (ASF). The most distinct changes occurred in specific microbe families associated with the consumption of a Western diet, characterized by high-fat and high-carbohydrate content^4-6,8.^

Microbes belonging to the family *Lachnospiraceae* (part of the phylum Firmicutes) were found to be elevated in infants fed formula, with the highest abundance in those who consumed ASF. We found that CPI for propionate production was increased in the formula-fed groups compared to infants who consumed expressed or pumped human milk. The family *Lachnospiraceae* is known to contain genes that contribute to the CPI of predicted propionate production,^20^ and it has been shown that increased abundance of *Lachnospiraceae* is associated with diets that are high in carbohydrates.^21^ Members of the family closely related to *Lachnospiraceae* are frequently found in the rumen of cows, goats and other ruminants,^22^ and are linked to obesity related conditions^23^ with the genus *Blautia* (a member of the *Lachnospiraceae* family) having been shown to be associated with obesity in Hispanic children.^24,25^ It follows that infants who consume either traditional lactose-based formula or lactose-reduced formula (which contains partially hydolyzed milk and whey protein concentrate solids) would have an increased abundance of microbes belonging to the *Lachnospiraceae* family.

A depletion of microbes belonging to *Bifidobacteriaceae* (a member of the phylum Actinobacteria) was observed in infants who consumed any formula, with greater decreases in ASF group. The gut microbiome of infants who consume human milk need to contain members of *Bifidobacteriaceae*, since specifically the species *B. longum* subsp. *infantis* is able to fully metabolize human milk oligosaccharides, one of the most abundant components of human milk.^26^ Also, we observed that CPI for lactose utilization, which is the most abundant milk sugar, was significantly higher in breastfed groups (BB and BP) compared to formula-fed groups (TF and ASF). Lactose utilization is one of the most common catabolic phenotype among *Bifidobacterium* spp., which could explain the increased predicted lactose utilization in the two groups consuming human milk and no formula.

In addition to the aforementioned microbes that were significantly different between groups of infants, we found that abundance of *Acidaminococcaceae* was higher in infants consuming lactose-reduced formula with added sugar compared to those who consume traditional formula. Currently, research examining the impact of diet on *Acidaminococcaceae* is sparse, with only a few studies in humans and in animal models. A member of the family *Acidaminococcaceae*, the genus *Phascolarctobacterium*, has been shown to be directly associated with the consumption of a Western diet, characterized by high fat and high carbohydrate content.^4-8^, Phascolarctobacterium has also been shown to be significantly higher in humans over the age of one compared to those less than one year old^27^

The importance of this study is bolstered by the inclusion of gut microbiota data at 1 month of age for use as baseline data, a timepoint in which a subset of the infants who are in the ASF and TF groups are exclusively breastfed. Controlling for microbiota at 1 month allowed for baseline shifts in the microbiome of the infants to be accounted for when examining differences by milk consumption type in the 6-month microbiome. Furthermore, the 1-month timepoint data was useful in demonstrating that these differences emerge with the introduction of infant formula. Additionally, we are able to distinguish between infants who consume human milk either directly from breast or from the bottle. This further division of the human milk consuming group allowed us to more confidently associate differential microbes with human milk as distinct from the act of breastfeeding. Our findings correspond with previous work that has shown infant outcomes differ in breastfed infants versus infants who consume human milk in a bottle.^28,29^

A limitation of the study is that the exact amount and type of milk consumed was not directly measured; however, formula type was assessed using maternal 24-hour dietary recalls. While it is likely that the type of formula consumed according to the dietary recall is indeed the primary formula type for the infant, we cannot be certain that the formula type consumed at the time of recall was consumed for a long time period. Also, while we were able to characterize the metabolic functions and phenotypes of the gut microbiome in this group of infants, this characterization is a prediction based on mapping of 16S data to the curated collection of metabolic phenotypes. It is important to note that this applied approach has a number of important limitations originating from both: (i) limited accuracy of phenotype projection from curated reference collection over 16S phylogenetic profiles; and (ii) the unknown extent of a predicted metabolic potential realization under specific conditions in the niche, a subject of complex regulation. Such functional predictions can be further refined by a combination with other *-omics* measurements and provide a starting point for focused experimental validation.^30^ This study provides new evidence that infants consuming lactose-reduced formula high in glucose have a more mature microbiome than infants consuming traditional formula and human milk. Future studies are necessary to determine the long-term effects on the developing infant gut microbiome and on the growth and development of infants who consume the lactose-reduced formula for an extended period of time.

## Methods

### Participants

This research examined data from 91 Hispanic infant-mother pairs enrolled in our ongoing prospective study (known as Mother’s Milk funded through DK110793) with the aim of determining the impact of breastfeeding and dietary sugar intake during the first two years of life on adiposity, possibly mediated by alterations in the developing gut microbiome. The inclusion and exclusion criteria for the Mother’s Milk study were previously reported in detail in Berger et al.^31^ Fecal samples to characterize the infants’ gut microbiome were collected at 1 month (to control for individual differences in microbiome) and 6 months of age, at which times infants’ length and weight were also measured. Mothers of the children completed several surveys to assess medical history and feeding behavior at 1 month and 6 months postpartum. Adjustment covariates extracted from these results included mother age at birth, pre-pregnancy BMI, mode of delivery, and mother current BMI as well as infant sex, age (days), and weight. To assess milk consumption type at 6 months of age, 24-hour diet recalls were performed in duplicate (first in person, second by telephone) for the infants by interviewing the mothers using the “multiple-pass” method and analyzed using the Nutrition Data System for Research software version 2014, developed by the Nutrition Coordinating Center, University of Minnesota. These dietary recalls allowed us to determine the milk consumption type and the frequency of milk consumption. Infants were divided into four groups based on feeding mode and type: (1) human milk directly from breast and no formula; (2) pumped human milk delivered from a bottle and no formula; (3) traditional lactose-based formula; and (4) lactose-reduced formula with added sugar as corn syrup solids.

### Ethics

Written parental consent for inclusion in these studies were obtained prior to any testing procedure for participants under 18 years of age. The University of Southern California and Children’s Hospital Los Angeles Institutional Review Board approved that these studies were conducted in accordance with the Declaration of Helsinki.

### Stool collection and DNA extraction/sequencing

Stool samples were collected using OMNIgene GUT collection kits (DNA Genotek, Ottawa, ON, CAN). Samples were stored at –80°C. 16S rRNA amplicon sequencing was used to characterize the microbiota. We prepared sample slurries according to the methods used by Flores et al.^32^ and subsequently extracted DNA using the PowerSoil‐htp 96 well soil DNA isolation kit (Mo Bio Laboratories, cat. no. 12955‐4) as recommended by the Earth Microbiome Project.^33^ Sequencing methods can be found in the Supplemental Material.

### 16S data processing

Sequence reads were demultiplexed with deML (https://grenaud.github.io/deML/). We used default parameters in DADA2^34(p2)^ (https://benjjneb.github.io/dada2/), available from QIIME2 software package for sequence read filtering, denoising, paired-read merging, removal of chimeras and obtaining of amplicon sequence variants (ASVs) with abundances. We assigned taxonomy to the obtained ASV sequences by blast with Ribosomal Database Project (RDP, version 11.5)^35^ and NCBI 16S rRNA gene combined databases using multitaxonomy approach (MTA). Further details of MTA approach can be found in the Supplemental Material. To calculate beta-diversity of the obtained ASVs, we used DEICODE, an Aitchison distance matrix that is robust to sparsity.^12^ ASVs were also collapsed into a total of 366 taxonomic groups (kingdom: n = 3; phylum: n = 13; class: n = 27; order: n = 40; family: n = 89; genus: n = 194; and species: n = 349). The raw 16S rRNA gene sequence reads used for this study are available on the NCBI Short Read Archive associated with the NCBI BioProject PRJNA589488.

### Prediction of metabolic functions and phenotypes

Functional gene assignments and metabolic reconstructions were performed using the SEED database and Web tools that allow subsystem-based analysis of ~6,000 bacterial genomes, including a subset of 2,660 reference human gut microbial genomes representing 690 species.^36^ The collection of curated metabolic subsystems includes (i) biosynthesis of essential nutrients (vitamins, amino acids), (ii) uptake and fermentation of carbohydrates including mono-, oligo-saccharides, sugar acids and alcohols, (iii) degradation of amino acids, and (iv) production of short chain fatty acids (SCFAs). Details on how 16S rRNA sequence reads were used to determine metabolic phenotypes from the metabolic reconstruction can be found in the Supplemental Methods. For each phenotype, we obtained a Community Phenotype Index (CPI) which represent a fractional representation (on a scale 0–100%) of microbial cells with a metabolic phenotype.

### Statistical analysis

To examine differences in diversity of gut microbiota, we conducted a perMANOVA (permutation multivariate analysis of variance) of beta diversities using 10,000 permutations. Pairwise linear discriminant analysis was used to determine taxa that are differentially abundant based on milk consumption types using the LEfSe algorithm on the Galaxy web application^33,34,37^ with the default parameters. Further, taxa that were considered to be differentially abundant between the ASF and BB groups were examined using linear regression models to determine if differences in abundances were statistically significant. Equation (1) was used to determine differences in microbial features (log-normalized^38^ relative abundance of taxonomic groups and CPI of predicted metabolic phenotypes) by infant’s milk consumption type.

(1) microbialFeature_i, 6 month_ = milk consumption type + mother age + maternal pre-pregnancy BMI+ delivery mode (vaginal/c-section) + maternal BMI + infant sex + infant age in days + infant weight + microbialFeature_i,1-month_ +e

The *p*-value from an ANOVA comparing these models to a reduced model that excludes “milk consumption type” as a dependent variable was used to determine significance of milk consumption type in the model. For microbiota found to be significantly associated with milk consumption type, the pairwise differences in the four milk consumption type groups were examined using the Tukey’s Honestly Significant Difference (HSD) test as a post-hoc analysis. Confidence intervals (95%) for false discovery rates were calculated using the fdrci R package^39^ with 1,000 permutations. Tests in which both *p* value was less than 0.05 and upper level of confidence interval for false discovery rate was less than 0.25 were considered to be statistically significant. All statistical analyses were performed in the R statistical computing language^40^ (version 3.6.0) and figures were generated using the ggplot2 R package.^41^ The code generated to conduct these statistical analyses can be found on GitHub using the link https://github.com/rbarner/infantMilkConsumption.

## Supplementary Material

Supplemental Material
