# Supplementary material for: Lactose-reduced infant formula with added corn syrup solids is associated with a distinct gut microbiota in Hispanic infants
Source: Gut Microbes. 2020 Sep 4;12(1):1813534. doi: 10.1080/19490976.2020.1813534 (PMC7524300; doi:10.1080/19490976.2020.1813534)
Supplement: Supplemental Material [file KGMI_A_1813534_SM3900.docx]

**Supplemental Methods**

*DNA extraction/sequencing*

The Earth Microbiome Project standard protocols (http://www.earthmicrobiome.org/protocols-and-standards/16s/) were employed with the barcoded primer set 515F-806R (515F:GTGCCAGCMGCCGCGGTAA, 806R:GGACTACHVGGGTWTCTAAT) that targets the V4 region of the bacterial (and archeal) 16S rRNA gene^20^. Paired-end, 2x150 base pairs, next-generation sequencing was performed using MiSeq Illumina platform (MiSeq Reagent Kit v2; Illumina Inc.; San Diego, CA, USA).

*16S Data processing*

MultiTaxonomy Approach (MTA) consisted of the following. Each representative sequence was aligned using NCBI BLAST toolkit (version 2.10.0) to the combined 16S rRNA gene reference database and the obtained alignments were sorted by the percentage of identity with maximum values denoted as M. We further collected and processed taxonomic assignments for identified 16S rRNA sequences with identities higher than the M‒(1‒M)/S threshold, where S=4 is a scaling parameter which controls the number of taxonomic descriptions accepted for multitaxonomy assignment. Resulting multitaxonomy assignments consisted of a ‘/’-separated list of taxonomic names with equal weights. According to their multitaxonomic descriptions, each samples`s ASV abundances were renormalized to account for 16S rRNA gene copy number in reference genomes using average 16S numbers at each taxonomic level provided by the rrnDB database^24^, and then finally converted to the corresponding relative values summing up to 1.

*Prediction of Metabolic Functions and Phenotypes*

Functional gene assignments and metabolic reconstructions were performed using the SEED database and Web tools that allow subsystem-based analysis of ~6,000 bacterial genomes, including a subset of 2,660 reference human gut microbial (HGM) genomes representing 690 species^26^. The subsystem-based approach for metabolic reconstruction combines protein similarity search, analysis of chromosomal gene clusters, and phylogenetic profiling^27^. The collection of curated metabolic subsystems includes (i) biosynthesis of essential nutrients (vitamins, amino acids), (ii) uptake and fermentation of carbohydrates including mono-, oligo-saccharides, sugar acids and alcohols, (iii) degradation of amino acids, and (iv) production of short chain fatty acids (SCFAs) projected the selected 2,660 HGM genomes. Using the collection of pathway-specific logical rules we obtained binary phenotype matrix (BPM) describing the inferred phenotypic features (nutrient requirements, utilization capabilities, metabolite production) for analyzed reference genomes. The obtained BPM for reference genomes was used to calculate a community phenotype matrix (CPM) for each taxonomically assigned ASV in 16S sample dataset as previously described^28^. At the next step, we renormalized ASV read counts by 16S rRNA gene copy number in microbial genomes using average 16S numbers at each taxonomic level provided by the rrnDB database^24^. We further calculated the Community Phenotype Index (CPI) for each metabolic pathway/metabolite using the renormalized table of ASV abundances and the respective CPM values. For each 16S sample and metabolic phenotype, CPI was calculated as the sum of CPM values for each taxon multiplied by their relative abundances. The obtained CPIs represent a fractional representation (on a scale 0-100%) of microbial cells with a metabolic phenotype (synthesis of essential nutrients such as amino acids and vitamins, utilization of sugars, degradation of amino acids, production of SCFAs).

**Supplemental Table 1: Macronutrient composition of three milk types according to Nutritional Data System for Research (NDSR).** Breastmilk composition is only an average as breast milk composition differs among women. Enfamil Infant Powder was the most popular choice of "traditional" formula in our group and Enfamil Gentlease was the only "gentle" formula in which the infants in our cohort consumed. We averaged the daily consumption of macronutrients within the three groups. While breastmilk was the sole milk source in the "BF"+"PF" group, the formula groups "TF" and "ASF" also had exposure to some breastfeeding. Each macronutrient was compared by "TF" vs "ASF" using a t-test. *P*-values from the t-tests are shown.

|  | **Breastmilk**  **(per 32 fl oz)** | **Enfamil Infant Powder** | **Enfamil Gentlease Powder** | **BF+PF (N=33)** | **TF (N=30)** | **ASF (N=29)** | ***p*-value ASF-TF** |
| --- | --- | --- | --- | --- | --- | --- | --- |
| Gram Amount of Food | 907.2 | 140.8 | 139.2 | 890.274 (476.625,1599) | 319.297 (81.358,1430.167) | 259.846 (66.407,964.969) | 0.675 |
| Energy (kcal) | 635.04 | 718.0664007 | 709.9271271 | 624.605 (333.637,1119.3) | 626.13 (414.933,1070.207) | 573.634 (338.673,956.252) | 0.409 |
| Total Fat (g) | 39.73346341 | 38.0129845 | 37.58479655 | 39.096 (20.876,70.031) | 34.576 (21.969,66.274) | 31.428 (17.93,50.628) | 0.416 |
| Total Saturated Fatty Acids [SFA]) (g) | 18.22618537 | 16.7212195 | 16.58510376 | 17.963 (9.575,32.123) | 15.348 (9.662,29.402) | 13.988 (7.913,22.344) | 0.449 |
| Total Monounsaturated Fatty Acids [MUFA]) (g) | 15.03992195 | 13.25635577 | 13.0970334 | 14.762 (7.902,26.507) | 12.372 (7.66,25.71) | 11.159 (6.248,17.64) | 0.411 |
| Total Polyunsaturated Fatty Acids [PUFA]) (g) | 4.50755122 | 6.574963255 | 6.67848086 | 4.439 (2.368,7.943) | 5.413 (3.76,7.904) | 5.137 (3.187,9) | 0.688 |
| ***SFA 4 0 (butyric acid) (g)*** | ***0*** | ***0.011825473*** | ***0*** | ***0 (0,0)*** | ***0.009 (0,0.018)*** | ***0.003 (0,0.006)*** | ***4.00E-10*** |
| SFA 6 0 (caproic acid) (g) | 0 | 0.047301894 | 0.046116315 | 0.005 (0,0.162) | 0.03 (0,0.051) | 0.03 (0.004,0.06) | 1 |
| SFA 8 0 (caprylic acid) (g) | 0 | 0.567622727 | 0.561780562 | 0.053 (0,1.799) | 0.372 (0.056,0.634) | 0.366 (0.047,0.756) | 0.995 |
| SFA 10 0 (capric acid) (g) | 0.571082927 | 0.461193466 | 0.448585971 | 0.577 (0.3,1.001) | 0.443 (0.266,0.934) | 0.392 (0.213,0.638) | 0.345 |
| SFA 12 0 (lauric acid) (g) | 2.324370732 | 3.393910889 | 3.341336626 | 2.282 (1.219,4.095) | 2.79 (1.947,3.969) | 2.585 (1.594,4.5) | 0.448 |
| SFA 14 0 (myristic acid) (g) | 2.914419512 | 1.478184185 | 1.433798151 | 2.857 (1.528,5.135) | 1.69 (0.854,4.675) | 1.444 (0.686,3.183) | 0.417 |
| SFA 16 0 (palmitic acid) (g) | 8.336546341 | 8.963708899 | 9.001066169 | 8.181 (4.379,14.69) | 7.829 (5.18,13.268) | 7.315 (4.293,12.12) | 0.6 |
| SFA 18 0 (stearic acid) (g) | 2.655219512 | 1.555049763 | 1.521838388 | 2.606 (1.395,4.68) | 1.674 (0.896,4.252) | 1.455 (0.726,2.918) | 0.416 |
| SFA 20 0 (arachidic acid) (g) | 0 | 0.029563684 | 0.025154354 | 0 (0,0.003) | 0.018 (0,0.031) | 0.017 (0.002,0.036) | 0.855 |
| SFA 22 0 (behenic acid) (g) | 0 | 0.088691051 | 0.083847845 | 0 (0,0.003) | 0.056 (0,0.096) | 0.054 (0.007,0.112) | 0.952 |
| MUFA 16 1 (palmitoleic acid) (g) | 1.169560976 | 0.065040104 | 0.062885884 | 1.149 (0.613,2.067) | 0.336 (0.04,1.828) | 0.242 (0.028,1.234) | 0.592 |
| MUFA 18 1 (oleic acid) (g) | 13.38146341 | 13.09079914 | 12.94610728 | 13.133 (7.03,23.582) | 11.851 (7.564,23.118) | 10.772 (6.176,17.436) | 0.416 |
| MUFA 20 1 (gadoleic acid) (g) | 0.364565854 | 0.088691051 | 0.088040237 | 0.356 (0.192,0.637) | 0.148 (0.054,0.567) | 0.121 (0.042,0.39) | 0.569 |
| PUFA 18 2 (linoleic acid) (g) | 3.392780488 | 5.634838114 | 5.705845857 | 3.345 (1.782,5.98) | 4.526 (3.05,6.287) | 4.31 (2.723,7.688) | 0.717 |
| PUFA 18 3 (linolenic acid) (g) | 0.472039024 | 0.579448201 | 0.612089269 | 0.465 (0.248,0.832) | 0.49 (0.335,0.789) | 0.48 (0.292,0.824) | 0.945 |
| PUFA 20 4 (arachidonic acid) (g) | 0.236019512 | 0.242422206 | 0.238966358 | 0.232 (0.124,0.416) | 0.216 (0.144,0.385) | 0.199 (0.115,0.324) | 0.481 |
| PUFA 22 6 (docosahexaenoic acid [DHA]) (g) | 0 | 0.124167472 | 0.121579375 | 0 (0,0) | 0.08 (0.006,0.136) | 0.079 (0.01,0.164) | 0.994 |
| TRANS 18 1 (trans octadecenoic acid) (g) | 2.176858537 | 0.041389157 | 0.033539138 | 2.134 (1.143,3.835) | 0.566 (0.02,3.393) | 0.401 (0.017,2.29) | 0.633 |
| TRANS 18 2 (trans octadecadienoic acid) (g) | 0.364565854 | 0.106429261 | 0.100617414 | 0.356 (0.192,0.637) | 0.16 (0.062,0.581) | 0.129 (0.048,0.392) | 0.48 |
| TRANS 16 1 (trans hexadecenoic acid) (g) | 0.088507317 | 0 | 0 | 0.087 (0.046,0.156) | 0.022 (0,0.138) | 0.015 (0,0.093) | 0.644 |
| Total Trans Fatty Acids (TRANS) (g) | 2.720546341 | 0.141905682 | 0.134156552 | 2.671 (1.43,4.797) | 0.77 (0.082,4.263) | 0.561 (0.064,2.871) | 0.616 |
| Polyunsaturated to Saturated Fat Ratio | 1.561521951 | 2.323705539 | 5.068602235 | 2.037 (0.864,3.211) | 2.677 (1.572,4.101) | 2.451 (1.441,4.232) | 0.31 |
| Cholesterol to Saturated Fatty Acid Index | 24.75886829 | 17.38344602 | 16.89114839 | 24.375 (13.006,43.641) | 17.412 (10.045,39.639) | 15.331 (8.058,27.438) | 0.375 |
| Cholesterol (mg) | 127.008 | 9.856532146 | 2.783748456 | 124.595 (66.727,223.86) | 38.202 (5.693,198.813) | 24.096 (1.329,133.69) | 0.366 |
| Total Carbohydrate (g) | 62.50513171 | 80.25357578 | 76.56146734 | 61.338 (32.838,110.175) | 67.852 (46.377,106.422) | 60.928 (36.524,103.128) | 0.231 |
| Available Carbohydrate (g) | 62.50513171 | 77.76431361 | 75.65591061 | 61.338 (32.838,110.175) | 66.101 (44.936,105.741) | 60.276 (36.091,101.904) | 0.347 |
| ***Total Sugars (g)**** | ***62.50513171*** | ***77.7465754*** | ***37.02720838*** | ***61.327 (32.838,110.175)*** | ***65.153 (44.929,105.667)*** | ***36.443 (17.663,68.795)*** | ***5.25E-10*** |
| ***Added Sugars (by Total Sugars) (g)***** | ***0*** | ***50.61893923*** | ***20.7271873*** | ***0.006 (0,0.188)*** | ***32.008 (4.994,56.514)*** | ***14.581 (1.745,27.92)*** | ***2.11E-09*** |
| ***Added Sugars (by Available Carbohydrate) (g)****** | ***0*** | ***50.61893923*** | ***63.753709*** | ***0.017 (0,0.576)*** | ***32.008 (4.994,56.514)*** | ***41.108 (5.367,85.872)*** | ***0.0483*** |
| ***Fructose (g)*** | ***0*** | ***0.011825473*** | ***0.096425022*** | ***0 (0,0)*** | ***0.011 (0,0.04)*** | ***0.06 (0.008,0.132)*** | ***3.99E-10*** |
| ***Galactose (g)*** | ***0*** | ***0.041389157*** | ***0.016769569*** | ***0 (0,0)*** | ***0.028 (0.003,0.05)*** | ***0.011 (0.001,0.02)*** | ***4.05E-10*** |
| ***Glucose (g)*** | ***0*** | ***0.112341998*** | ***12.16632232*** | ***0.003 (0,0.109)*** | ***0.081 (0.004,0.26)*** | ***7.506 (1.024,16.388)*** | ***3.99E-10*** |
| ***Lactose (g)*** | ***62.50513171*** | ***76.67637005*** | ***15.3148089*** | ***61.321 (32.838,110.175)*** | ***64.449 (44.307,105.63)*** | ***23.016 (7.304,66.967)*** | ***3.99E-10*** |
| ***Maltose (g)*** | ***0*** | ***0*** | ***8.602788905*** | ***0.002 (0,0.079)*** | ***0.008 (0,0.156)*** | ***5.304 (0.724,11.588)*** | ***3.99E-10*** |
| ***Sucrose (g)*** | ***0*** | ***0.011825473*** | ***0.834286058*** | ***0 (0,0)*** | ***0.011 (0,0.04)*** | ***0.516 (0.07,1.128)*** | ***3.99E-10*** |
| ***Glycemic Index (glucose reference)*** | ***41*** | ***42.845*** | ***91.338*** | ***43.725 (41,133.659)*** | ***58.402 (41.923,105.688)*** | ***101.346 (67.091,136.819)*** | ***<2E-16*** |
| ***Glycemic Index (bread reference)*** | ***58.63*** | ***61.207*** | ***130.483*** | ***62.523 (58.63,191)*** | ***83.454 (59.929,151.041)*** | ***144.8 (95.845,195.513)*** | ***<2E-16*** |
| ***Glycemic Load (glucose reference)*** | ***25.62708293*** | ***33.31827153*** | ***69.10320152*** | ***25.16 (13.463,45.175)*** | ***28.56 (19.25,43.541)*** | ***48.256 (25.563,93.08)*** | ***1.79E-08*** |
| ***Glycemic Load (bread reference)*** | ***36.64835122*** | ***47.59753076*** | ***98.7182604*** | ***35.98 (19.253,64.597)*** | ***40.808 (27.504,62.258)*** | ***68.943 (36.546,132.972)*** | ***1.79E-08*** |
| ***Total Dietary Fiber (g)*** | ***0*** | ***2.489262168*** | ***0.905556727*** | ***0 (0,0)*** | ***1.751 (0,4.849)*** | ***0.65 (0.076,1.246)*** | ***5.44E-10*** |
| ***Soluble Dietary Fiber (g)*** | ***0*** | ***2.489262168*** | ***0.402469656*** | ***0 (0,0)*** | ***1.751 (0,4.849)*** | ***0.34 (0.034,0.815)*** | ***3.99E-10*** |
| ***Insoluble Dietary Fiber (g)*** | ***0*** | ***0*** | ***0.498894678*** | ***0 (0,0)*** | ***0 (0,0)*** | ***0.308 (0.042,0.676)*** | ***3.99E-10*** |
| Total Protein (g) | 9.34595122 | 14.22013186 | 16.28744391 | 9.296 (4.908,16.471) | 11.784 (7.911,16.249) | 12.175 (7.769,21.936) | 0.869 |
| ***Animal Protein (g)*** | ***9.34595122*** | ***14.22013186*** | ***5.793886095*** | ***9.296 (4.908,16.471)*** | ***11.784 (7.911,16.249)*** | ***5.704 (2.763,10.307)*** | ***3.99E-10*** |
| ***Vegetable Protein (g)*** | ***0*** | ***0*** | ***10.4977502*** | ***0 (0,0)*** | ***0 (0,0)*** | ***6.471 (0.884,14.136)*** | ***3.99E-10*** |
| Tryptophan (g) | 0.153834146 | 0.230596733 | 0.251543535 | 0.154 (0.079,0.273) | 0.189 (0.13,0.264) | 0.191 (0.12,0.34) | 0.986 |
| Threonine (g) | 0.419356098 | 0.804132197 | 0.89297955 | 0.416 (0.218,0.741) | 0.625 (0.4,0.894) | 0.65 (0.387,1.2) | 0.815 |
| Isoleucine (g) | 0.507863415 | 0.928299668 | 1.018751318 | 0.506 (0.265,0.897) | 0.731 (0.478,1.04) | 0.748 (0.467,1.368) | 0.93 |
| Leucine (g) | 0.861892683 | 1.57870071 | 1.735650393 | 0.86 (0.451,1.521) | 1.253 (0.808,1.76) | 1.278 (0.794,2.344) | 0.948 |
| Lysine (g) | 0.61955122 | 1.058379877 | 1.165485047 | 0.618 (0.326,1.092) | 0.863 (0.562,1.309) | 0.866 (0.558,1.576) | 0.998 |
| Methionine (g) | 0.189658537 | 0.295636837 | 0.33539138 | 0.19 (0.1,0.338) | 0.248 (0.162,0.422) | 0.25 (0.161,0.452) | 0.992 |
| Cystine (g) | 0.170692683 | 0.183294839 | 0.201234828 | 0.17 (0.09,0.299) | 0.159 (0.103,0.282) | 0.161 (0.096,0.272) | 0.983 |
| Phenylalanine (g) | 0.419356098 | 0.591273674 | 0.654013192 | 0.416 (0.218,0.741) | 0.505 (0.341,0.798) | 0.496 (0.312,0.88) | 0.962 |
| Tyrosine (g) | 0.478360976 | 0.691790199 | 0.750438213 | 0.477 (0.252,0.845) | 0.576 (0.394,0.827) | 0.571 (0.357,1.012) | 0.993 |
| Valine (g) | 0.571082927 | 0.946037878 | 1.031328494 | 0.566 (0.3,1.001) | 0.763 (0.505,1.052) | 0.769 (0.493,1.388) | 0.993 |
| Arginine (g) | 0.389853659 | 0.337025994 | 0.377315303 | 0.388 (0.203,0.689) | 0.33 (0.198,0.668) | 0.311 (0.179,0.504) | 0.74 |
| Histidine (g) | 0.206517073 | 0.295636837 | 0.33539138 | 0.206 (0.108,0.364) | 0.255 (0.17,0.456) | 0.253 (0.161,0.452) | 0.997 |
| Alanine (g) | 0.324526829 | 0.650401041 | 0.712706683 | 0.323 (0.17,0.572) | 0.501 (0.319,0.724) | 0.519 (0.306,0.956) | 0.853 |
| Aspartic Acid (g) | 0.743882927 | 1.395405871 | 1.530223173 | 0.741 (0.389,1.313) | 1.092 (0.706,1.556) | 1.124 (0.689,2.064) | 0.898 |
| Glutamic Acid (g) | 1.523590244 | 2.814462688 | 3.202987682 | 1.522 (0.8,2.691) | 2.264 (1.448,3.406) | 2.339 (1.417,4.312) | 0.873 |
| Glycine (g) | 0.236019512 | 0.295636837 | 0.33539138 | 0.233 (0.124,0.416) | 0.254 (0.171,0.402) | 0.258 (0.161,0.452) | 0.957 |
| Proline (g) | 0.743882927 | 1.265325662 | 1.391874228 | 0.741 (0.389,1.313) | 1.033 (0.675,1.534) | 1.032 (0.663,1.876) | 1 |
| Serine (g) | 0.389853659 | 0.733179356 | 0.809131705 | 0.39 (0.203,0.689) | 0.588 (0.376,0.893) | 0.592 (0.362,1.088) | 0.994 |

*Total sugar includes monosaccharides and disaccharides

**Added Sugars (by Total Sugars) (g) includes added monosaccharides and disaccharides.

***Added Sugars (by Available Carbohydrate) (g) includes monosaccharides, disaccharides and polysaccharides.

**Supplemental Table 2:** Order-level taxa association with milk consumption type.

|  |  |  |  |  |  | **BP-BB** | | **TF-BB** | | **TF-BP** | | **ASF-BB** | | **ASF-BP** | | **ASF-TF** | |
| --- | --- | --- | --- | --- | --- | --- | --- | --- | --- | --- | --- | --- | --- | --- | --- | --- | --- |
| **Taxa** | **BB (n=14)**  **Mean (SD)** | **BP (n=20) Mean (SD)** | **TF (n=30) Mean (SD)** | **ASF (n=27) Mean (SD)** | **ANOVA**  ***p*-value** | **Mean Difference** | ***p*-value** | **Mean Difference** | ***p*-value** | **Mean Difference** | ***p*-value** | **Mean Difference** | ***p*-value** | **Mean Difference** | ***p*-value** | **Mean Difference** | ***p*-value** |
| Bacteroidales | 3.1 (1.1) | 3.3 (1.1) | 3.7 (0.85) | 3.8 (0.72) | 0.0668 | 0.23 | 0.86 | 0.82 | 0.017 | 0.59 | 0.085 | 0.72 | 0.041 | 0.5 | 0.19 | -0.096 | 0.97 |
| Clostridiales | 2.6 (0.61) | 3 (0.67) | 3.4 (0.47) | 3.5 (0.42) | 0.000137 | 0.46 | 0.083 | 0.77 | 0.00024 | 0.31 | 0.23 | 0.89 | 1.70E-05 | 0.43 | 0.044 | 0.12 | 0.83 |
| Enterobacterales | 3.7 (0.53) | 3.3 (0.74) | 3.2 (0.44) | 3 (0.65) | 0.00284 | -0.51 | 0.048 | -0.58 | 0.0099 | -0.064 | 0.98 | -0.71 | 0.00097 | -0.19 | 0.64 | -0.13 | 0.81 |
| Bifidobacteriales | 3.7 (0.63) | 3.6 (0.6) | 3.2 (0.81) | 2.8 (1.1) | 0.00291 | -0.22 | 0.89 | -0.71 | 0.07 | -0.49 | 0.25 | -1.1 | 0.0023 | -0.83 | 0.011 | -0.34 | 0.46 |
| Veillonellales | 2.8 (0.8) | 3.1 (0.76) | 3 (0.84) | 2.8 (0.95) | 0.683 | 0.25 | 0.86 | 0.089 | 0.99 | -0.16 | 0.93 | 0.041 | 1 | -0.21 | 0.86 | -0.048 | 1 |
| Burkholderiales | 0.46 (0.94) | 1.1 (1.5) | 1.8 (1.6) | 1.7 (1.5) | 0.216 | 0.93 | 0.27 | 1.4 | 0.025 | 0.44 | 0.74 | 1.4 | 0.022 | 0.45 | 0.72 | 0.012 | 1 |
| Verrucomicrobiales | 0.29 (0.84) | 0.14 (0.45) | 0.78 (1) | 1 (1.4) | 0.048 | 0.096 | 0.99 | 0.82 | 0.069 | 0.73 | 0.087 | 0.94 | 0.027 | 0.85 | 0.032 | 0.12 | 0.97 |
| Lactobacillales | 2.3 (0.57) | 2.2 (0.47) | 1.9 (0.8) | 1.8 (0.71) | 0.157 | -0.23 | 0.76 | -0.62 | 0.028 | -0.39 | 0.22 | -0.58 | 0.042 | -0.35 | 0.29 | 0.037 | 1 |
| Acidaminococcales | 0.17 (0.64) | 0.19 (0.61) | 0.23 (0.67) | 0.97 (1.4) | 0.00497 | 0.22 | 0.91 | 0.26 | 0.83 | 0.04 | 1 | 0.97 | 0.011 | 0.75 | 0.041 | 0.71 | 0.026 |
| Erysipelotrichales | 0.62 (1) | 0.63 (0.91) | 1.3 (0.9) | 1.6 (0.7) | 0.00725 | 0.074 | 1 | 0.81 | 0.042 | 0.74 | 0.044 | 0.95 | 0.011 | 0.88 | 0.011 | 0.14 | 0.94 |
| Pasteurellales | 1.5 (0.89) | 0.84 (0.85) | 1.1 (1.1) | 0.96 (0.75) | 0.43 | -0.65 | 0.24 | -0.45 | 0.47 | 0.19 | 0.91 | -0.59 | 0.24 | 0.053 | 1 | -0.14 | 0.95 |
| Desulfovibrionales | 0.19 (0.72) | 0.37 (0.8) | 0.59 (1) | 1 (1.1) | 0.0624 | 0.22 | 0.9 | 0.29 | 0.77 | 0.064 | 1 | 0.84 | 0.028 | 0.62 | 0.11 | 0.55 | 0.11 |
| Bacteroidales_Lactobacillales | 0 (0) | 0.25 (0.65) | 0.082 (0.31) | 0.23 (0.68) | 0.584 | 0.3 | 0.48 | 0.095 | 0.96 | -0.2 | 0.65 | 0.23 | 0.63 | -0.071 | 0.98 | 0.13 | 0.83 |
| Coriobacteriales | 1.1 (1) | 0.78 (1) | 1.3 (1) | 1 (0.93) | 0.0609 | -0.53 | 0.46 | 0.2 | 0.93 | 0.74 | 0.088 | -0.17 | 0.96 | 0.37 | 0.63 | -0.37 | 0.52 |
| Actinomycetales | 0.56 (0.7) | 0.79 (0.92) | 0.32 (0.52) | 0.13 (0.37) | 0.117 | 0.068 | 0.99 | -0.28 | 0.47 | -0.34 | 0.21 | -0.47 | 0.065 | -0.54 | 0.013 | -0.2 | 0.57 |
| Bacillales | 0.28 (0.57) | 0.29 (0.54) | 0.38 (0.59) | 0.45 (0.67) | 0.928 | -0.012 | 1 | 0.078 | 0.98 | 0.09 | 0.97 | 0.15 | 0.9 | 0.16 | 0.85 | 0.068 | 0.98 |
| Eggerthellales | 0.49 (0.7) | 0.58 (0.7) | 0.3 (0.54) | 0.55 (0.63) | 0.804 | -0.045 | 1 | -0.18 | 0.84 | -0.13 | 0.91 | 0.019 | 1 | 0.064 | 0.99 | 0.2 | 0.67 |
| Pseudomonadales | 0.066 (0.25) | 0.03 (0.14) | 0.37 (0.65) | 0.4 (0.5) | 0.094 | -0.035 | 1 | 0.28 | 0.3 | 0.31 | 0.14 | 0.33 | 0.16 | 0.36 | 0.062 | 0.051 | 0.98 |
| Fusobacteriales | 0.042 (0.16) | 0.029 (0.13) | 0.32 (0.68) | 0.064 (0.23) | 0.162 | -0.011 | 1 | 0.21 | 0.39 | 0.22 | 0.28 | 0.019 | 1 | 0.03 | 0.99 | -0.19 | 0.29 |
| Micrococcales | 0.66 (0.72) | 0.34 (0.62) | 0.27 (0.58) | 0.12 (0.36) | 0.0152 | -0.39 | 0.16 | -0.57 | 0.0063 | -0.19 | 0.62 | -0.59 | 0.0047 | -0.2 | 0.57 | -0.012 | 1 |
| Campylobacterales | 0.42 (0.69) | 0.3 (0.55) | 0.56 (0.73) | 0.43 (0.64) | 0.92 | -0.11 | 0.97 | -0.046 | 1 | 0.06 | 0.99 | -0.022 | 1 | 0.084 | 0.97 | 0.024 | 1 |
| Neisseriales | 0.068 (0.25) | 0.17 (0.43) | 0.23 (0.47) | 0.087 (0.32) | 0.516 | 0.13 | 0.78 | 0.13 | 0.72 | 0.0018 | 1 | 0.014 | 1 | -0.12 | 0.74 | -0.12 | 0.65 |

**Supplemental Table 3**: Family-level taxa association with milk consumption type.

|  |  |  |  |  |  | **BP-BB** | | **TF-BB** | | **TF-BP** | | **ASF-BB** | | **ASF-BP** | | **ASF-TF** | |
| --- | --- | --- | --- | --- | --- | --- | --- | --- | --- | --- | --- | --- | --- | --- | --- | --- | --- |
| **Taxa** | **BB (n=14) Mean (SD)** | **BP (n=20) Mean (SD)** | **TF (n=30) Mean (SD)** | **ASF (n=27) Mean (SD)** | **ANOVA *p*-value** | **Mean Difference** | ***p*-value** | **Mean Difference** | ***p*-value** | **Mean Difference** | ***p*-value** | **Mean Difference** | ***p*-value** | **Mean Difference** | ***p*-value** | **Mean Difference** | ***p*-value** |
| Ruminococcaceae_{unclassified} | 0.23 (0.61) | 0.77 (1.2) | 2.1 (1.1) | 2.4 (1) | 2.80E-08 | 0.82 | 0.14 | 2.2 | 2.10E-07 | 1.3 | 0.00045 | 2.3 | 2.30E-08 | 1.5 | 5.90E-05 | 0.17 | 0.93 |
| Lachnospiraceae | 2 (1) | 2.5 (1) | 2.9 (0.6) | 3.2 (0.45) | 2.51E-05 | 0.66 | 0.043 | 0.89 | 0.001 | 0.23 | 0.67 | 1.2 | 3.30E-06 | 0.58 | 0.032 | 0.34 | 0.25 |
| Peptostreptococcaceae | 0.56 (0.69) | 0.91 (1) | 1.3 (0.64) | 1.6 (0.51) | 0.000172 | 0.15 | 0.93 | 0.72 | 0.012 | 0.57 | 0.039 | 0.95 | 0.00043 | 0.8 | 0.0014 | 0.23 | 0.6 |
| Enterobacteriaceae | 3.7 (0.53) | 3.3 (0.74) | 3.2 (0.44) | 3 (0.65) | 0.00259 | -0.52 | 0.046 | -0.59 | 0.0087 | -0.07 | 0.97 | -0.71 | 0.00094 | -0.19 | 0.65 | -0.12 | 0.84 |
| Bifidobacteriaceae | 3.7 (0.63) | 3.6 (0.6) | 3.2 (0.81) | 2.8 (1.1) | 0.00291 | -0.22 | 0.89 | -0.71 | 0.07 | -0.49 | 0.25 | -1.1 | 0.0023 | -0.83 | 0.011 | -0.34 | 0.46 |
| Lactobacillaceae | 0.99 (0.8) | 1.4 (0.83) | 0.45 (0.65) | 0.6 (0.7) | 0.00345 | 0.33 | 0.6 | -0.65 | 0.046 | -0.99 | 0.00023 | -0.47 | 0.23 | -0.8 | 0.0034 | 0.19 | 0.79 |
| Acidaminococcaceae | 0.17 (0.64) | 0.19 (0.61) | 0.23 (0.67) | 0.97 (1.4) | 0.00497 | 0.22 | 0.91 | 0.26 | 0.83 | 0.04 | 1 | 0.97 | 0.011 | 0.75 | 0.041 | 0.71 | 0.026 |
| Erysipelotrichaceae | 0.62 (1) | 0.63 (0.91) | 1.3 (0.9) | 1.6 (0.7) | 0.00725 | 0.074 | 1 | 0.81 | 0.042 | 0.74 | 0.044 | 0.95 | 0.011 | 0.88 | 0.011 | 0.14 | 0.94 |
| Micrococcaceae | 0.66 (0.72) | 0.34 (0.62) | 0.27 (0.58) | 0.12 (0.36) | 0.0152 | -0.39 | 0.16 | -0.57 | 0.0063 | -0.19 | 0.62 | -0.59 | 0.0047 | -0.2 | 0.57 | -0.012 | 1 |
| Atopobiaceae | 0.67 (0.94) | 0.3 (0.74) | 0.16 (0.5) | 0.25 (0.63) | 0.0281 | -0.61 | 0.022 | -0.62 | 0.011 | -0.00085 | 1 | -0.47 | 0.075 | 0.14 | 0.84 | 0.15 | 0.79 |
| Coriobacteriaceae | 0.55 (0.93) | 0.57 (0.91) | 1.3 (1) | 0.81 (0.91) | 0.0357 | -0.041 | 1 | 0.79 | 0.086 | 0.83 | 0.037 | 0.22 | 0.91 | 0.26 | 0.82 | -0.57 | 0.15 |
| Akkermansiaceae | 0.29 (0.84) | 0.14 (0.45) | 0.78 (1) | 1 (1.4) | 0.048 | 0.096 | 0.99 | 0.82 | 0.069 | 0.73 | 0.087 | 0.94 | 0.027 | 0.85 | 0.032 | 0.12 | 0.97 |
| Bacteroidaceae | 2.6 (1.5) | 2.9 (1.4) | 3.2 (1.1) | 3.5 (0.96) | 0.0501 | 0.48 | 0.57 | 0.94 | 0.038 | 0.46 | 0.46 | 1 | 0.024 | 0.52 | 0.36 | 0.051 | 1 |
| Moraxellaceae | 0.066 (0.25) | 0.03 (0.14) | 0.28 (0.52) | 0.4 (0.5) | 0.06 | -0.035 | 1 | 0.26 | 0.31 | 0.29 | 0.14 | 0.33 | 0.12 | 0.36 | 0.041 | 0.071 | 0.93 |
| Desulfovibrionaceae | 0.19 (0.72) | 0.37 (0.8) | 0.59 (1) | 1 (1.1) | 0.0624 | 0.22 | 0.9 | 0.29 | 0.77 | 0.064 | 1 | 0.84 | 0.028 | 0.62 | 0.11 | 0.55 | 0.11 |
| Actinomycetaceae | 0.56 (0.7) | 0.79 (0.92) | 0.32 (0.52) | 0.13 (0.37) | 0.117 | 0.068 | 0.99 | -0.28 | 0.47 | -0.34 | 0.21 | -0.47 | 0.065 | -0.54 | 0.013 | -0.2 | 0.57 |
| Streptococcaceae | 1.9 (0.86) | 1.7 (0.59) | 1.6 (1) | 1.3 (0.9) | 0.142 | -0.38 | 0.62 | -0.63 | 0.13 | -0.25 | 0.77 | -0.74 | 0.053 | -0.36 | 0.51 | -0.11 | 0.96 |
| {unclassified:Flintibacter} | 0 (0) | 0.22 (0.56) | 0.42 (0.86) | 0.73 (1.1) | 0.159 | 0.26 | 0.85 | 0.48 | 0.38 | 0.22 | 0.86 | 0.73 | 0.071 | 0.47 | 0.31 | 0.25 | 0.72 |
| Fusobacteriaceae | 0.042 (0.16) | 0.029 (0.13) | 0.32 (0.68) | 0.064 (0.23) | 0.162 | -0.011 | 1 | 0.21 | 0.39 | 0.22 | 0.28 | 0.019 | 1 | 0.03 | 0.99 | -0.19 | 0.29 |
| Oscillospiraceae | 0 (0) | 0.062 (0.28) | 0.33 (0.7) | 0.61 (1.1) | 0.163 | 0.073 | 0.99 | 0.39 | 0.42 | 0.31 | 0.52 | 0.61 | 0.076 | 0.54 | 0.095 | 0.22 | 0.69 |
| Tannerellaceae | 1.1 (1) | 1.6 (1.4) | 2 (1.5) | 2 (1.4) | 0.178 | 0.57 | 0.57 | 0.96 | 0.092 | 0.39 | 0.72 | 0.83 | 0.18 | 0.26 | 0.9 | -0.13 | 0.98 |
| Sutterellaceae | 0.46 (0.94) | 1.1 (1.5) | 1.8 (1.6) | 1.7 (1.5) | 0.217 | 0.93 | 0.27 | 1.4 | 0.025 | 0.44 | 0.74 | 1.4 | 0.022 | 0.45 | 0.72 | 0.01 | 1 |
| Rikenellaceae | 0.088 (0.33) | 0.3 (0.78) | 0.44 (0.85) | 0.92 (1.3) | 0.231 | 0.26 | 0.85 | 0.41 | 0.49 | 0.15 | 0.94 | 0.82 | 0.028 | 0.57 | 0.15 | 0.41 | 0.3 |
| Morganellaceae | 0.13 (0.48) | 0.29 (0.72) | 0.21 (0.57) | 0.24 (0.54) | 0.252 | 0.34 | 0.32 | 0.24 | 0.56 | -0.1 | 0.93 | 0.24 | 0.56 | -0.1 | 0.93 | -0.0023 | 1 |
| Ruminococcaceae | 1.1 (0.76) | 1.5 (0.95) | 1.9 (1.2) | 2 (0.99) | 0.278 | 0.49 | 0.58 | 0.88 | 0.07 | 0.39 | 0.63 | 0.86 | 0.075 | 0.37 | 0.66 | -0.017 | 1 |
| Muribaculaceae | 0 (0) | 0.17 (0.42) | 0.066 (0.25) | 0.12 (0.36) | 0.341 | 0.13 | 0.58 | 0.04 | 0.98 | -0.094 | 0.71 | 0.12 | 0.58 | -0.012 | 1 | 0.083 | 0.71 |
| Enterococcaceae | 1.1 (1) | 1.1 (0.93) | 0.57 (0.75) | 1 (0.86) | 0.389 | -0.044 | 1 | -0.42 | 0.51 | -0.38 | 0.53 | -0.0098 | 1 | 0.034 | 1 | 0.42 | 0.34 |
| Pasteurellaceae | 1.5 (0.89) | 0.84 (0.85) | 1.1 (1.1) | 0.96 (0.75) | 0.43 | -0.65 | 0.24 | -0.45 | 0.47 | 0.19 | 0.91 | -0.59 | 0.24 | 0.053 | 1 | -0.14 | 0.95 |
| Neisseriaceae | 0.068 (0.25) | 0.17 (0.43) | 0.23 (0.47) | 0.087 (0.32) | 0.516 | 0.13 | 0.78 | 0.13 | 0.72 | 0.0018 | 1 | 0.014 | 1 | -0.12 | 0.74 | -0.12 | 0.65 |
| Bacteroidaceae_Lactobacillaceae | 0 (0) | 0.25 (0.65) | 0.082 (0.31) | 0.23 (0.68) | 0.584 | 0.3 | 0.48 | 0.095 | 0.96 | -0.2 | 0.65 | 0.23 | 0.63 | -0.071 | 0.98 | 0.13 | 0.83 |
| Prevotellaceae | 2 (0.75) | 1.8 (0.26) | 2.1 (0.79) | 2 (0.72) | 0.664 | -0.18 | 0.88 | 0.11 | 0.96 | 0.29 | 0.49 | -0.062 | 0.99 | 0.12 | 0.94 | -0.17 | 0.77 |
| Veillonellaceae | 2.8 (0.8) | 3.1 (0.76) | 3 (0.84) | 2.8 (0.95) | 0.683 | 0.25 | 0.86 | 0.089 | 0.99 | -0.16 | 0.93 | 0.041 | 1 | -0.21 | 0.86 | -0.048 | 1 |
| Clostridiaceae_Ruminococcaceae | 0 (0) | 0.15 (0.37) | 0.15 (0.38) | 0.18 (0.45) | 0.751 | 0.17 | 0.58 | 0.17 | 0.54 | -0.0052 | 1 | 0.18 | 0.47 | 0.008 | 1 | 0.013 | 1 |
| Eggerthellaceae | 0.49 (0.7) | 0.58 (0.7) | 0.3 (0.54) | 0.55 (0.63) | 0.804 | -0.045 | 1 | -0.18 | 0.84 | -0.13 | 0.91 | 0.019 | 1 | 0.064 | 0.99 | 0.2 | 0.67 |
| Clostridiaceae | 1.5 (1.2) | 1.8 (0.97) | 1.9 (0.98) | 1.8 (0.92) | 0.92 | 0.19 | 0.95 | 0.26 | 0.86 | 0.069 | 1 | 0.19 | 0.94 | -0.0071 | 1 | -0.076 | 0.99 |
| Eubacteriaceae | 0.17 (0.43) | 0.12 (0.53) | 0.23 (0.61) | 0.32 (0.79) | 0.936 | 0.053 | 1 | 0.17 | 0.87 | 0.12 | 0.94 | 0.23 | 0.73 | 0.18 | 0.82 | 0.058 | 0.99 |
| Campylobacteraceae | 0.16 (0.45) | 0.087 (0.27) | 0.28 (0.6) | 0.15 (0.43) | 0.947 | -0.067 | 0.97 | -0.028 | 1 | 0.039 | 0.99 | -0.024 | 1 | 0.043 | 0.98 | 0.0044 | 1 |
| Helicobacteraceae | 0.3 (0.6) | 0.21 (0.52) | 0.32 (0.59) | 0.29 (0.55) | 0.95 | -0.08 | 0.98 | -0.059 | 0.99 | 0.022 | 1 | -0.04 | 1 | 0.041 | 1 | 0.019 | 1 |
| Staphylococcaceae | 0.28 (0.57) | 0.24 (0.53) | 0.33 (0.58) | 0.41 (0.65) | 0.978 | -0.012 | 1 | 0.018 | 1 | 0.03 | 1 | 0.11 | 0.95 | 0.12 | 0.92 | 0.089 | 0.95 |

**Supplemental Table 4**: Genus-level taxa association with milk consumption type.

|  |  |  |  |  |  | ***BP-BB*** | | ***TF-BB*** | | ***TF-BP*** | | **ASF-BB** | | ***ASF-BP*** | | ***ASF-TF*** | |
| --- | --- | --- | --- | --- | --- | --- | --- | --- | --- | --- | --- | --- | --- | --- | --- | --- | --- |
| **Taxa** | **BB (n=14) Mean (SD)** | **BP (n=20) Mean (SD)** | **TF (n=30) Mean (SD)** | **ASF (n=27) Mean (SD)** | **ANOVA *p*-value** | **Mean Difference** | ***p*-value** | **Mean Difference** | ***p*-value** | **Mean Difference** | ***p*-value** | **Mean Difference** | ***p*-value** | **Mean Difference** | ***p*-value** | **Mean Difference** | ***p*-value** |
| Flavonifractor1 | 0.23 (0.61) | 0.72 (1.2) | 2.1 (1.1) | 2.3 (1.1) | 7.57E-08 | 0.75 | 0.23 | 2.2 | 6.10E-07 | 1.4 | 0.00044 | 2.3 | 1.80E-07 | 1.5 | 0.00014 | 0.096 | 0.99 |
| Lachnoclostridium | 0.65 (0.74) | 1.2 (0.99) | 1.9 (0.96) | 2.4 (0.72) | 1.85E-05 | 0.54 | 0.29 | 1.3 | 5.70E-05 | 0.79 | 0.015 | 1.7 | 4.40E-07 | 1.1 | 0.00019 | 0.34 | 0.45 |
| Clostridioides | 0.19 (0.52) | 0.34 (0.75) | 0.67 (0.69) | 1 (0.76) | 0.000229 | 0.032 | 1 | 0.52 | 0.11 | 0.49 | 0.095 | 0.84 | 0.0024 | 0.81 | 0.0013 | 0.31 | 0.33 |
| Blautia | 1.7 (1.3) | 2 (1.3) | 2.5 (0.83) | 2.9 (0.47) | 0.000324 | 0.55 | 0.31 | 0.96 | 0.0089 | 0.41 | 0.44 | 1.4 | 9.70E-05 | 0.8 | 0.021 | 0.39 | 0.36 |
| Bifidobacterium | 3.7 (0.63) | 3.6 (0.6) | 3.2 (0.81) | 2.8 (1.1) | 0.00291 | -0.22 | 0.89 | -0.71 | 0.07 | -0.49 | 0.25 | -1.1 | 0.0023 | -0.83 | 0.011 | -0.34 | 0.46 |
| Lactobacillus | 0.99 (0.8) | 1.4 (0.83) | 0.45 (0.65) | 0.6 (0.7) | 0.00345 | 0.33 | 0.6 | -0.65 | 0.046 | -0.99 | 0.00023 | -0.47 | 0.23 | -0.8 | 0.0034 | 0.19 | 0.79 |
| Collinsella | 0.53 (0.9) | 0.46 (0.84) | 1.2 (1) | 0.8 (0.9) | 0.0126 | -0.15 | 0.97 | 0.8 | 0.069 | 0.95 | 0.0096 | 0.23 | 0.89 | 0.38 | 0.56 | -0.57 | 0.13 |
| Phascolarctobacterium | 0.17 (0.64) | 0.12 (0.55) | 0.23 (0.67) | 0.76 (1.3) | 0.0146 | 0.14 | 0.96 | 0.26 | 0.79 | 0.12 | 0.97 | 0.76 | 0.04 | 0.62 | 0.086 | 0.5 | 0.13 |
| Rothia | 0.66 (0.72) | 0.34 (0.62) | 0.27 (0.58) | 0.12 (0.36) | 0.0152 | -0.39 | 0.16 | -0.57 | 0.0063 | -0.19 | 0.62 | -0.59 | 0.0047 | -0.2 | 0.57 | -0.012 | 1 |
| Anaerotruncus | 0 (0) | 0.073 (0.33) | 0.22 (0.46) | 0.54 (0.82) | 0.0254 | 0.086 | 0.97 | 0.26 | 0.5 | 0.17 | 0.74 | 0.54 | 0.022 | 0.45 | 0.043 | 0.28 | 0.24 |
| Citrobacter | 0 (0) | 0.52 (0.98) | 0.45 (0.8) | 0.76 (1.1) | 0.0297 | 0.43 | 0.47 | 0.41 | 0.44 | -0.02 | 1 | 0.76 | 0.029 | 0.33 | 0.53 | 0.35 | 0.37 |
| Escherichia | 3.5 (0.77) | 3 (1.1) | 2.8 (0.63) | 2.3 (0.98) | 0.0315 | -0.59 | 0.24 | -0.75 | 0.054 | -0.16 | 0.93 | -1.2 | 0.00063 | -0.58 | 0.13 | -0.42 | 0.27 |
| Erysipelatoclostridium | 0.51 (1) | 0.63 (0.91) | 1.3 (0.88) | 1.4 (0.89) | 0.0378 | 0.19 | 0.95 | 0.86 | 0.046 | 0.66 | 0.12 | 0.83 | 0.052 | 0.64 | 0.13 | -0.023 | 1 |
| Streptococcus | 1.9 (0.86) | 1.7 (0.59) | 1.5 (1) | 1.1 (0.87) | 0.0417 | -0.38 | 0.62 | -0.71 | 0.067 | -0.34 | 0.57 | -0.89 | 0.012 | -0.52 | 0.2 | -0.18 | 0.87 |
| Atopobium | 0.67 (0.94) | 0.3 (0.74) | 0.16 (0.5) | 0.2 (0.59) | 0.0458 | -0.61 | 0.021 | -0.62 | 0.01 | -0.00085 | 1 | -0.52 | 0.037 | 0.094 | 0.95 | 0.095 | 0.93 |
| Akkermansia | 0.29 (0.84) | 0.14 (0.45) | 0.78 (1) | 1 (1.4) | 0.048 | 0.096 | 0.99 | 0.82 | 0.069 | 0.73 | 0.087 | 0.94 | 0.027 | 0.85 | 0.032 | 0.12 | 0.97 |
| Bacteroides | 2.6 (1.5) | 2.9 (1.4) | 3.2 (1.1) | 3.5 (0.96) | 0.0501 | 0.48 | 0.57 | 0.94 | 0.038 | 0.46 | 0.46 | 1 | 0.024 | 0.52 | 0.36 | 0.051 | 1 |
| Acinetobacter | 0.066 (0.25) | 0.03 (0.14) | 0.28 (0.52) | 0.4 (0.5) | 0.06 | -0.035 | 1 | 0.26 | 0.31 | 0.29 | 0.14 | 0.33 | 0.12 | 0.36 | 0.041 | 0.071 | 0.93 |
| Lactococcus | 0 (0) | 0 (0) | 0.26 (0.65) | 0.5 (0.83) | 0.0875 | -7.90E-16 | 1 | 0.3 | 0.43 | 0.3 | 0.35 | 0.5 | 0.062 | 0.5 | 0.035 | 0.2 | 0.61 |
| Tyzzerella | 0.041 (0.15) | 0.57 (0.96) | 0.53 (0.85) | 0.8 (0.87) | 0.0956 | 0.63 | 0.19 | 0.45 | 0.41 | -0.18 | 0.9 | 0.76 | 0.044 | 0.13 | 0.96 | 0.31 | 0.53 |
| Actinomyces | 0.56 (0.7) | 0.79 (0.92) | 0.32 (0.52) | 0.13 (0.37) | 0.117 | 0.068 | 0.99 | -0.28 | 0.47 | -0.34 | 0.21 | -0.47 | 0.065 | -0.54 | 0.013 | -0.2 | 0.57 |
| Bilophila | 0.19 (0.72) | 0.3 (0.77) | 0.55 (1) | 0.86 (1.2) | 0.138 | 0.15 | 0.96 | 0.24 | 0.83 | 0.091 | 0.98 | 0.65 | 0.1 | 0.5 | 0.22 | 0.41 | 0.28 |
| Caproiciproducens_Neglecta | 0.38 (0.63) | 0.34 (0.61) | 0.1 (0.32) | 0.13 (0.34) | 0.148 | -0.014 | 1 | -0.29 | 0.29 | -0.27 | 0.26 | -0.28 | 0.33 | -0.26 | 0.3 | 0.014 | 1 |
| Intestinibacter | 0.33 (0.61) | 0.5 (0.82) | 0.84 (0.76) | 0.83 (0.82) | 0.156 | 0.085 | 0.99 | 0.48 | 0.28 | 0.4 | 0.37 | 0.47 | 0.29 | 0.39 | 0.39 | -0.011 | 1 |
| Fusobacterium | 0.042 (0.16) | 0.029 (0.13) | 0.32 (0.68) | 0.064 (0.23) | 0.162 | -0.011 | 1 | 0.21 | 0.39 | 0.22 | 0.28 | 0.019 | 1 | 0.03 | 0.99 | -0.19 | 0.29 |
| Oscillibacter | 0 (0) | 0.062 (0.28) | 0.33 (0.7) | 0.61 (1.1) | 0.163 | 0.073 | 0.99 | 0.39 | 0.42 | 0.31 | 0.52 | 0.61 | 0.076 | 0.54 | 0.095 | 0.22 | 0.69 |
| Parabacteroides | 1.1 (1) | 1.6 (1.4) | 2 (1.5) | 2 (1.4) | 0.178 | 0.57 | 0.57 | 0.96 | 0.092 | 0.39 | 0.72 | 0.83 | 0.18 | 0.26 | 0.9 | -0.13 | 0.98 |
| Ruthenibacterium | 0 (0) | 0.26 (0.65) | 0.29 (0.68) | 0.62 (1) | 0.188 | 0.3 | 0.74 | 0.33 | 0.62 | 0.031 | 1 | 0.62 | 0.12 | 0.31 | 0.6 | 0.28 | 0.58 |
| Hungatella | 0.34 (0.91) | 0.45 (0.96) | 0.74 (1) | 1.1 (1) | 0.212 | 0.16 | 0.97 | 0.44 | 0.57 | 0.28 | 0.81 | 0.71 | 0.16 | 0.55 | 0.29 | 0.27 | 0.76 |
| {unclassified:[Eubacterium] rectale} | 0.1 (0.38) | 0.21 (0.66) | 0.28 (0.86) | 0.65 (1.1) | 0.227 | 0.044 | 1 | 0.091 | 0.99 | 0.047 | 1 | 0.54 | 0.25 | 0.5 | 0.25 | 0.45 | 0.23 |
| Alistipes | 0.088 (0.33) | 0.3 (0.78) | 0.44 (0.85) | 0.92 (1.3) | 0.231 | 0.26 | 0.85 | 0.41 | 0.49 | 0.15 | 0.94 | 0.82 | 0.028 | 0.57 | 0.15 | 0.41 | 0.3 |
| Anaerostipes_Lachnoclostridium | 0 (0) | 0.4 (0.73) | 0.38 (0.66) | 0.44 (0.58) | 0.263 | 0.47 | 0.17 | 0.39 | 0.26 | -0.085 | 0.97 | 0.44 | 0.16 | -0.031 | 1 | 0.055 | 0.99 |
| Ruminococcus | 0.26 (0.45) | 0.36 (0.84) | 0.71 (1.1) | 0.48 (0.97) | 0.276 | 0.14 | 0.97 | 0.56 | 0.27 | 0.42 | 0.45 | 0.27 | 0.81 | 0.13 | 0.97 | -0.29 | 0.64 |
| Dorea | 0 (0) | 0.19 (0.6) | 0.24 (0.54) | 0.44 (0.96) | 0.295 | 0.22 | 0.81 | 0.27 | 0.64 | 0.052 | 0.99 | 0.44 | 0.22 | 0.22 | 0.71 | 0.17 | 0.8 |
| Faecalimonas | 0 (0) | 0.067 (0.3) | 0.38 (0.67) | 0.4 (0.72) | 0.298 | 0.079 | 0.98 | 0.38 | 0.18 | 0.3 | 0.3 | 0.4 | 0.14 | 0.32 | 0.23 | 0.024 | 1 |
| Proteus | 0 (0) | 0.19 (0.6) | 0.16 (0.51) | 0.24 (0.54) | 0.312 | 0.23 | 0.61 | 0.18 | 0.7 | -0.04 | 0.99 | 0.24 | 0.48 | 0.017 | 1 | 0.057 | 0.98 |
| Muribaculum | 0 (0) | 0.17 (0.42) | 0.066 (0.25) | 0.12 (0.36) | 0.341 | 0.13 | 0.58 | 0.04 | 0.98 | -0.094 | 0.71 | 0.12 | 0.58 | -0.012 | 1 | 0.083 | 0.71 |
| Roseburia | 0.083 (0.31) | 0.44 (0.96) | 0.55 (0.9) | 0.51 (0.92) | 0.354 | 0.42 | 0.57 | 0.55 | 0.27 | 0.13 | 0.97 | 0.42 | 0.5 | -0.00082 | 1 | -0.13 | 0.95 |
| Megasphaera | 0.3 (0.62) | 0.75 (1.1) | 0.45 (1.1) | 0.45 (0.86) | 0.362 | 0.45 | 0.56 | -0.029 | 1 | -0.48 | 0.36 | 0.13 | 0.98 | -0.32 | 0.69 | 0.16 | 0.93 |
| Klebsiella | 1.5 (1.3) | 2.1 (0.94) | 2.3 (1) | 2.3 (1) | 0.371 | 0.44 | 0.64 | 0.68 | 0.21 | 0.24 | 0.88 | 0.78 | 0.11 | 0.34 | 0.7 | 0.1 | 0.98 |
| Enterococcus | 1.1 (1) | 1.1 (0.93) | 0.57 (0.75) | 1 (0.86) | 0.389 | -0.044 | 1 | -0.42 | 0.51 | -0.38 | 0.53 | -0.0098 | 1 | 0.034 | 1 | 0.42 | 0.34 |
| Haemophilus | 1.5 (0.89) | 0.84 (0.85) | 1.1 (1.1) | 0.96 (0.75) | 0.43 | -0.65 | 0.24 | -0.45 | 0.47 | 0.19 | 0.91 | -0.59 | 0.24 | 0.053 | 1 | -0.14 | 0.95 |
| Clostridium | 1.4 (1.1) | 1.7 (0.96) | 1.5 (1.1) | 1.2 (1.1) | 0.511 | 0.13 | 0.99 | 0.0016 | 1 | -0.13 | 0.98 | -0.33 | 0.81 | -0.46 | 0.52 | -0.33 | 0.69 |
| Neisseria | 0.068 (0.25) | 0.17 (0.43) | 0.23 (0.47) | 0.087 (0.32) | 0.516 | 0.13 | 0.78 | 0.13 | 0.72 | 0.0018 | 1 | 0.014 | 1 | -0.12 | 0.74 | -0.12 | 0.65 |
| Fusicatenibacter | 0.076 (0.28) | 0.32 (0.83) | 0.29 (0.77) | 0.25 (0.62) | 0.522 | 0.29 | 0.69 | 0.26 | 0.72 | -0.034 | 1 | 0.17 | 0.9 | -0.12 | 0.95 | -0.088 | 0.97 |
| Prevotella | 2 (0.75) | 1.8 (0.26) | 2.1 (0.79) | 1.9 (0.67) | 0.573 | -0.18 | 0.88 | 0.11 | 0.96 | 0.29 | 0.48 | -0.13 | 0.93 | 0.048 | 1 | -0.24 | 0.52 |
| Bacteroides_Lactobacillus | 0 (0) | 0.25 (0.65) | 0.082 (0.31) | 0.23 (0.68) | 0.584 | 0.3 | 0.48 | 0.095 | 0.96 | -0.2 | 0.65 | 0.23 | 0.63 | -0.071 | 0.98 | 0.13 | 0.83 |
| Veillonella | 2.8 (0.8) | 3 (0.85) | 2.8 (0.99) | 2.5 (1.3) | 0.623 | 0.094 | 0.99 | -0.055 | 1 | -0.15 | 0.97 | -0.27 | 0.88 | -0.36 | 0.69 | -0.21 | 0.88 |
| Sutterella | 0.46 (0.94) | 0.94 (1.5) | 1.3 (1.6) | 1.3 (1.5) | 0.628 | 0.77 | 0.39 | 0.89 | 0.2 | 0.12 | 0.99 | 0.92 | 0.17 | 0.16 | 0.98 | 0.033 | 1 |
| Anaerostipes | 0.066 (0.25) | 0.15 (0.47) | 0.29 (0.67) | 0.32 (0.74) | 0.671 | 0.11 | 0.96 | 0.27 | 0.55 | 0.16 | 0.83 | 0.25 | 0.59 | 0.14 | 0.86 | -0.015 | 1 |
| Lachnospira | 0.19 (0.72) | 0.18 (0.6) | 0.28 (0.79) | 0.51 (1) | 0.757 | -0.0021 | 1 | 0.018 | 1 | 0.02 | 1 | 0.3 | 0.72 | 0.3 | 0.65 | 0.28 | 0.61 |
| Faecalibacterium | 0.85 (0.86) | 1.2 (0.9) | 1.2 (1.1) | 1.2 (0.97) | 0.919 | 0.37 | 0.73 | 0.31 | 0.77 | -0.053 | 1 | 0.24 | 0.88 | -0.12 | 0.98 | -0.071 | 0.99 |
| Eubacterium | 0.17 (0.43) | 0.12 (0.53) | 0.23 (0.61) | 0.32 (0.79) | 0.936 | 0.053 | 1 | 0.17 | 0.87 | 0.12 | 0.94 | 0.23 | 0.73 | 0.18 | 0.82 | 0.058 | 0.99 |
| Raoultella | 0.091 (0.34) | 0.29 (0.62) | 0.17 (0.65) | 0.17 (0.6) | 0.938 | 0.098 | 0.95 | 0.0022 | 1 | -0.096 | 0.93 | 0.07 | 0.98 | -0.028 | 1 | 0.068 | 0.96 |
| Eggerthella | 0.49 (0.7) | 0.54 (0.62) | 0.3 (0.54) | 0.49 (0.6) | 0.945 | -0.045 | 1 | -0.18 | 0.84 | -0.13 | 0.9 | -0.041 | 1 | 0.0032 | 1 | 0.14 | 0.86 |
| Campylobacter | 0.16 (0.45) | 0.087 (0.27) | 0.28 (0.6) | 0.15 (0.43) | 0.947 | -0.067 | 0.97 | -0.028 | 1 | 0.039 | 0.99 | -0.024 | 1 | 0.043 | 0.98 | 0.0044 | 1 |
| Helicobacter | 0.3 (0.6) | 0.21 (0.52) | 0.32 (0.59) | 0.29 (0.55) | 0.95 | -0.08 | 0.98 | -0.059 | 0.99 | 0.022 | 1 | -0.04 | 1 | 0.041 | 1 | 0.019 | 1 |
| Staphylococcus | 0.28 (0.57) | 0.24 (0.53) | 0.33 (0.58) | 0.41 (0.65) | 0.978 | -0.012 | 1 | 0.018 | 1 | 0.03 | 1 | 0.11 | 0.95 | 0.12 | 0.92 | 0.089 | 0.95 |

**Supplemental Table 5:** Species-level taxa association with milk consumption type.

|  |  |  |  |  |  | ***BP-BB*** | | ***TF-BB*** | | ***TF-BP*** | | ***ASF-BB*** | | ***ASF-BP*** | | ***ASF-TF*** | |
| --- | --- | --- | --- | --- | --- | --- | --- | --- | --- | --- | --- | --- | --- | --- | --- | --- | --- |
| **Taxa** | **BB (n=14) Mean (SD)** | **BP (n=20) Mean (SD)** | **TF (n=30) Mean (SD)** | **ASF (n=27) Mean (SD)** | **ANOVA *p*-value** | **Mean Difference** | ***p*-value** | **Mean Difference** | ***p*-value** | **Mean Difference** | ***p*-value** | **Mean Difference** | ***p*-value** | **Mean Difference** | ***p*-value** | **Mean Difference** | ***p*-value** |
| Flavonifractor plautii1 | 0.23 (0.61) | 0.72 (1.2) | 2.1 (1.1) | 2.3 (1.1) | 7.57E-08 | 0.75 | 0.23 | 2.2 | 6.10E-07 | 1.4 | 0.00044 | 2.3 | 1.80E-07 | 1.5 | 0.00014 | 0.096 | 0.99 |
| [Clostridium] clostridioforme | 0.099 (0.37) | 0.31 (0.76) | 0.82 (0.96) | 1.7 (0.99) | 1.43E-05 | 0.26 | 0.85 | 0.77 | 0.055 | 0.51 | 0.25 | 1.6 | 4.90E-06 | 1.3 | 3.00E-05 | 0.83 | 0.0053 |
| Lactobacillus rhamnosus | 0.48 (0.8) | 0.88 (0.86) | 0 (0) | 0.045 (0.24) | 4.71E-05 | 0.34 | 0.29 | -0.52 | 0.021 | -0.85 | 6.30E-06 | -0.47 | 0.04 | -0.81 | 1.60E-05 | 0.045 | 0.99 |
| Clostridioides difficile | 0.19 (0.52) | 0.34 (0.75) | 0.67 (0.69) | 1 (0.76) | 0.000229 | 0.032 | 1 | 0.52 | 0.11 | 0.49 | 0.095 | 0.84 | 0.0024 | 0.81 | 0.0013 | 0.31 | 0.33 |
| Streptococcus mitis_oralis | 0.19 (0.48) | 0.38 (0.68) | 0.12 (0.45) | 0 (0) | 0.00408 | 0.24 | 0.4 | -0.15 | 0.73 | -0.39 | 0.02 | -0.2 | 0.47 | -0.44 | 0.0051 | -0.057 | 0.96 |
| Blautia obeum_wexlerae | 0.29 (0.61) | 0.38 (0.86) | 1.3 (1.2) | 1.6 (1.4) | 0.00685 | 0.13 | 0.99 | 1 | 0.058 | 0.88 | 0.078 | 1.3 | 0.0076 | 1.2 | 0.009 | 0.29 | 0.8 |
| [Clostridium] symbiosum | 0 (0) | 0.22 (0.67) | 0.2 (0.64) | 0.97 (1.2) | 0.0072 | 0.25 | 0.82 | 0.23 | 0.83 | -0.024 | 1 | 0.97 | 0.0034 | 0.71 | 0.026 | 0.74 | 0.0068 |
| Bacteroides uniformis | 0.61 (1.1) | 0.86 (1.2) | 1.1 (1.4) | 1.7 (1.6) | 0.00892 | 0.29 | 0.91 | 0.51 | 0.57 | 0.22 | 0.93 | 1 | 0.062 | 0.71 | 0.21 | 0.49 | 0.43 |
| Bifidobacterium bifidum | 2.6 (1.4) | 1.8 (1.7) | 1.3 (1.4) | 0.99 (1.3) | 0.00918 | -1.2 | 0.08 | -1.7 | 0.0034 | -0.44 | 0.73 | -1.8 | 0.0013 | -0.56 | 0.55 | -0.12 | 0.99 |
| [Ruminococcus] gnavus | 1.5 (1.3) | 1.7 (1.3) | 2.1 (0.87) | 2.4 (0.73) | 0.0134 | 0.28 | 0.87 | 0.74 | 0.14 | 0.46 | 0.47 | 0.94 | 0.036 | 0.66 | 0.16 | 0.2 | 0.89 |
| [Clostridium] innocuum | 0 (0) | 0.22 (0.67) | 0.31 (0.43) | 0.71 (0.78) | 0.0143 | 0.26 | 0.68 | 0.35 | 0.34 | 0.096 | 0.96 | 0.71 | 0.006 | 0.45 | 0.093 | 0.36 | 0.16 |
| Rothia mucilaginosa | 0.66 (0.72) | 0.34 (0.62) | 0.27 (0.58) | 0.12 (0.36) | 0.0152 | -0.39 | 0.16 | -0.57 | 0.0063 | -0.19 | 0.62 | -0.59 | 0.0047 | -0.2 | 0.57 | -0.012 | 1 |
| Phascolarctobacterium faecium | 0.17 (0.64) | 0.12 (0.55) | 0.23 (0.67) | 0.72 (1.3) | 0.0203 | 0.14 | 0.96 | 0.26 | 0.79 | 0.12 | 0.97 | 0.72 | 0.058 | 0.57 | 0.12 | 0.46 | 0.19 |
| [Clostridium] aldenense | 0 (0) | 0.085 (0.38) | 0.54 (0.87) | 0.81 (1) | 0.0216 | 0.1 | 0.98 | 0.62 | 0.066 | 0.52 | 0.11 | 0.81 | 0.0076 | 0.71 | 0.012 | 0.19 | 0.77 |
| Citrobacter freundii | 0 (0) | 0.44 (0.95) | 0.45 (0.8) | 0.76 (1.1) | 0.0218 | 0.33 | 0.66 | 0.41 | 0.43 | 0.074 | 0.99 | 0.76 | 0.027 | 0.43 | 0.3 | 0.35 | 0.36 |
| Anaerotruncus colihominis | 0 (0) | 0.073 (0.33) | 0.22 (0.46) | 0.54 (0.82) | 0.0254 | 0.086 | 0.97 | 0.26 | 0.5 | 0.17 | 0.74 | 0.54 | 0.022 | 0.45 | 0.043 | 0.28 | 0.24 |
| Escherichia coli | 3.5 (0.77) | 3 (1.1) | 2.8 (0.63) | 2.3 (0.98) | 0.0315 | -0.59 | 0.24 | -0.75 | 0.054 | -0.16 | 0.93 | -1.2 | 0.00063 | -0.58 | 0.13 | -0.42 | 0.27 |
| Lactococcus lactis | 0 (0) | 0 (0) | 0.17 (0.46) | 0.47 (0.77) | 0.0394 | -5.20E-16 | 1 | 0.2 | 0.62 | 0.2 | 0.55 | 0.47 | 0.025 | 0.47 | 0.012 | 0.27 | 0.18 |
| Collinsella aerofaciens | 0.53 (0.9) | 0.46 (0.84) | 1.1 (0.96) | 0.8 (0.9) | 0.0432 | -0.15 | 0.97 | 0.66 | 0.17 | 0.81 | 0.033 | 0.23 | 0.89 | 0.38 | 0.56 | -0.43 | 0.34 |
| Atopobium parvulum | 0.67 (0.94) | 0.3 (0.74) | 0.16 (0.5) | 0.2 (0.59) | 0.0458 | -0.61 | 0.021 | -0.62 | 0.01 | -0.00085 | 1 | -0.52 | 0.037 | 0.094 | 0.95 | 0.095 | 0.93 |
| Akkermansia muciniphila | 0.29 (0.84) | 0.14 (0.45) | 0.78 (1) | 1 (1.4) | 0.048 | 0.096 | 0.99 | 0.82 | 0.069 | 0.73 | 0.087 | 0.94 | 0.027 | 0.85 | 0.032 | 0.12 | 0.97 |
| Veillonella atypica_dispar | 1.5 (1.1) | 1.3 (1.2) | 2.2 (1.1) | 1.7 (1.3) | 0.048 | -0.12 | 0.99 | 0.52 | 0.54 | 0.63 | 0.28 | 0.2 | 0.95 | 0.32 | 0.8 | -0.32 | 0.74 |
| Bacteroides caccae | 0.21 (0.55) | 0.64 (1.3) | 1.1 (1.4) | 1.1 (1.5) | 0.0729 | 0.53 | 0.62 | 0.93 | 0.099 | 0.4 | 0.69 | 0.88 | 0.12 | 0.36 | 0.76 | -0.049 | 1 |
| Prevotella bivia | 0.12 (0.3) | 0.42 (0.54) | 0.067 (0.27) | 0.066 (0.24) | 0.0749 | 0.24 | 0.27 | -0.051 | 0.97 | -0.29 | 0.053 | -0.062 | 0.96 | -0.3 | 0.04 | -0.011 | 1 |
| Bacteroides ovatus | 0.67 (1.1) | 1 (1.5) | 1.2 (1.3) | 0.79 (1.2) | 0.0791 | 0.31 | 0.8 | 0.61 | 0.21 | 0.3 | 0.71 | 0.073 | 1 | -0.24 | 0.84 | -0.54 | 0.15 |
| Clostridium perfringens | 0.64 (1) | 0.53 (0.89) | 0.24 (0.57) | 0.11 (0.42) | 0.0805 | -0.17 | 0.9 | -0.55 | 0.084 | -0.38 | 0.28 | -0.58 | 0.063 | -0.41 | 0.22 | -0.026 | 1 |
| Erysipelatoclostridium ramosum | 0.51 (1) | 0.41 (0.76) | 1.2 (0.95) | 1.1 (0.93) | 0.0811 | -0.065 | 1 | 0.74 | 0.12 | 0.8 | 0.046 | 0.55 | 0.33 | 0.62 | 0.17 | -0.18 | 0.9 |
| Parabacteroides distasonis | 0.46 (1.2) | 1.1 (1.6) | 1.7 (1.6) | 1.5 (1.6) | 0.0867 | 0.79 | 0.42 | 1.4 | 0.026 | 0.57 | 0.55 | 1.1 | 0.12 | 0.27 | 0.92 | -0.3 | 0.85 |
| Tyzzerella nexilis | 0 (0) | 0.37 (0.95) | 0.47 (0.83) | 0.8 (0.86) | 0.105 | 0.44 | 0.49 | 0.49 | 0.32 | 0.051 | 1 | 0.8 | 0.031 | 0.36 | 0.52 | 0.3 | 0.55 |
| Actinomyces lingnae | 0.23 (0.48) | 0.087 (0.39) | 0.2 (0.47) | 0.081 (0.29) | 0.118 | -0.25 | 0.21 | -0.063 | 0.95 | 0.19 | 0.31 | -0.17 | 0.48 | 0.081 | 0.88 | -0.11 | 0.68 |
| Sutterella massiliensis_stercoricanis | 0.46 (0.94) | 0.51 (1.1) | 0.54 (1.2) | 0.9 (1.4) | 0.119 | 0.27 | 0.88 | -0.0057 | 1 | -0.27 | 0.81 | 0.57 | 0.33 | 0.3 | 0.76 | 0.57 | 0.16 |
| Veillonella dispar | 1.3 (1.4) | 1.8 (1.4) | 0.9 (1.4) | 1.3 (1.6) | 0.132 | 0.47 | 0.81 | -0.46 | 0.79 | -0.93 | 0.17 | -0.028 | 1 | -0.5 | 0.68 | 0.43 | 0.7 |
| Bilophila wadsworthia | 0.19 (0.72) | 0.3 (0.77) | 0.55 (1) | 0.86 (1.2) | 0.138 | 0.15 | 0.96 | 0.24 | 0.83 | 0.091 | 0.98 | 0.65 | 0.1 | 0.5 | 0.22 | 0.41 | 0.28 |
| Bacteroides thetaiotaomicron | 0.64 (1.4) | 0.51 (1.1) | 1.3 (1.3) | 0.92 (1.3) | 0.148 | -0.085 | 1 | 0.7 | 0.37 | 0.79 | 0.21 | 0.23 | 0.95 | 0.32 | 0.85 | -0.47 | 0.54 |
| Caproiciproducens galactitolivorans_Neglecta timonensis | 0.38 (0.63) | 0.34 (0.61) | 0.1 (0.32) | 0.13 (0.34) | 0.148 | -0.014 | 1 | -0.29 | 0.29 | -0.27 | 0.26 | -0.28 | 0.33 | -0.26 | 0.3 | 0.014 | 1 |
| Streptococcus thermophilus | 1.2 (0.84) | 1.2 (0.69) | 1.1 (0.94) | 0.71 (0.83) | 0.153 | -0.18 | 0.94 | -0.32 | 0.68 | -0.14 | 0.95 | -0.58 | 0.17 | -0.4 | 0.41 | -0.26 | 0.66 |
| Intestinibacter bartlettii | 0.33 (0.61) | 0.5 (0.82) | 0.84 (0.76) | 0.83 (0.82) | 0.156 | 0.085 | 0.99 | 0.48 | 0.28 | 0.4 | 0.37 | 0.47 | 0.29 | 0.39 | 0.39 | -0.011 | 1 |
| Oscillibacter ruminantium | 0 (0) | 0.062 (0.28) | 0.33 (0.7) | 0.61 (1.1) | 0.163 | 0.073 | 0.99 | 0.39 | 0.42 | 0.31 | 0.52 | 0.61 | 0.076 | 0.54 | 0.095 | 0.22 | 0.69 |
| [Clostridium] celerecrescens_[Clostridium] indolis_[Desulfotomaculum] guttoideum | 0 (0) | 0.063 (0.28) | 0.63 (1) | 0.41 (0.89) | 0.171 | 0.074 | 0.99 | 0.72 | 0.045 | 0.65 | 0.052 | 0.41 | 0.42 | 0.34 | 0.51 | -0.31 | 0.5 |
| [Clostridium] scindens | 0.38 (0.54) | 0.5 (0.57) | 0.28 (0.47) | 0.34 (0.5) | 0.187 | 0.051 | 0.99 | -0.22 | 0.56 | -0.27 | 0.3 | -0.071 | 0.97 | -0.12 | 0.86 | 0.15 | 0.69 |
| Ruthenibacterium lactatiformans | 0 (0) | 0.26 (0.65) | 0.29 (0.68) | 0.62 (1) | 0.188 | 0.3 | 0.74 | 0.33 | 0.62 | 0.031 | 1 | 0.62 | 0.12 | 0.31 | 0.6 | 0.28 | 0.58 |
| Clostridium celatum_disporicum_saudiense | 0 (0) | 0.24 (0.55) | 0.31 (0.8) | 0.07 (0.25) | 0.201 | 0.16 | 0.84 | 0.36 | 0.19 | 0.2 | 0.61 | 0.07 | 0.98 | -0.09 | 0.95 | -0.29 | 0.19 |
| Hungatella hathewayi | 0.34 (0.91) | 0.45 (0.96) | 0.74 (1) | 1.1 (1) | 0.212 | 0.16 | 0.97 | 0.44 | 0.57 | 0.28 | 0.81 | 0.71 | 0.16 | 0.55 | 0.29 | 0.27 | 0.76 |
| [Eubacterium] rectale | 0.1 (0.38) | 0.21 (0.66) | 0.28 (0.86) | 0.65 (1.1) | 0.227 | 0.044 | 1 | 0.091 | 0.99 | 0.047 | 1 | 0.54 | 0.25 | 0.5 | 0.25 | 0.45 | 0.23 |
| Bifidobacterium dentium | 0.53 (1.1) | 0.89 (1.3) | 0.3 (0.85) | 0.33 (0.81) | 0.231 | 0.37 | 0.75 | -0.32 | 0.79 | -0.7 | 0.13 | -0.24 | 0.9 | -0.61 | 0.21 | 0.083 | 0.99 |
| Bifidobacterium longum | 3 (1.4) | 2.8 (1.5) | 2.7 (1.2) | 2.2 (1.4) | 0.252 | -0.46 | 0.8 | -0.3 | 0.92 | 0.16 | 0.98 | -0.93 | 0.2 | -0.47 | 0.69 | -0.63 | 0.35 |
| Blautia hansenii_producta | 0 (0) | 0.061 (0.27) | 0.28 (0.65) | 0.46 (0.86) | 0.256 | 0.072 | 0.98 | 0.32 | 0.31 | 0.25 | 0.46 | 0.46 | 0.063 | 0.39 | 0.1 | 0.14 | 0.77 |
| Anaerostipes caccae_[Clostridium] indolis | 0 (0) | 0.4 (0.73) | 0.38 (0.66) | 0.44 (0.58) | 0.263 | 0.47 | 0.17 | 0.39 | 0.26 | -0.085 | 0.97 | 0.44 | 0.16 | -0.031 | 1 | 0.055 | 0.99 |
| Clostridium butyricum | 0.16 (0.59) | 0 (0) | 0.57 (0.96) | 0.29 (0.61) | 0.264 | -0.17 | 0.9 | 0.39 | 0.33 | 0.56 | 0.046 | 0.12 | 0.95 | 0.29 | 0.49 | -0.26 | 0.49 |
| Ruminococcus faecis | 0.091 (0.34) | 0.22 (0.72) | 0.46 (0.96) | 0.31 (0.77) | 0.288 | 0.16 | 0.94 | 0.43 | 0.38 | 0.26 | 0.7 | 0.21 | 0.86 | 0.045 | 1 | -0.22 | 0.74 |
| Faecalimonas umbilicata | 0 (0) | 0.067 (0.3) | 0.38 (0.67) | 0.4 (0.72) | 0.298 | 0.079 | 0.98 | 0.38 | 0.18 | 0.3 | 0.3 | 0.4 | 0.14 | 0.32 | 0.23 | 0.024 | 1 |
| Klebsiella oxytoca | 0.33 (0.86) | 1 (1.1) | 1.2 (1.3) | 1.1 (1.3) | 0.302 | 0.62 | 0.49 | 0.83 | 0.17 | 0.21 | 0.94 | 0.92 | 0.11 | 0.29 | 0.85 | 0.084 | 0.99 |
| Bacteroides massiliensis | 0.062 (0.23) | 0.12 (0.37) | 0.62 (1.3) | 0.44 (1.1) | 0.306 | 0.074 | 1 | 0.6 | 0.2 | 0.53 | 0.24 | 0.37 | 0.61 | 0.3 | 0.71 | -0.23 | 0.78 |
| Proteus mirabilis | 0 (0) | 0.19 (0.6) | 0.16 (0.51) | 0.24 (0.54) | 0.312 | 0.23 | 0.61 | 0.18 | 0.7 | -0.04 | 0.99 | 0.24 | 0.48 | 0.017 | 1 | 0.057 | 0.98 |
| Bifidobacterium kashiwanohense_pseudocatenulatum | 1.3 (1.1) | 1.2 (1.4) | 0.99 (1.3) | 0.67 (1) | 0.336 | -0.47 | 0.72 | -0.31 | 0.87 | 0.16 | 0.97 | -0.78 | 0.23 | -0.31 | 0.83 | -0.47 | 0.48 |
| Bacteroides vulgatus | 1.9 (1.3) | 2.1 (1.1) | 2.2 (1.2) | 2.6 (1.4) | 0.339 | 0.55 | 0.48 | 0.49 | 0.52 | -0.067 | 1 | 0.8 | 0.12 | 0.24 | 0.88 | 0.31 | 0.71 |
| Muribaculum intestinale | 0 (0) | 0.17 (0.42) | 0.066 (0.25) | 0.12 (0.36) | 0.341 | 0.13 | 0.58 | 0.04 | 0.98 | -0.094 | 0.71 | 0.12 | 0.58 | -0.012 | 1 | 0.083 | 0.71 |
| Bifidobacterium adolescentis | 0.22 (0.84) | 0.62 (1.1) | 0.92 (1.3) | 1.1 (1.4) | 0.359 | 0.49 | 0.69 | 0.82 | 0.2 | 0.33 | 0.82 | 0.86 | 0.16 | 0.37 | 0.76 | 0.036 | 1 |
| Megasphaera micronuciformis | 0.24 (0.6) | 0.49 (0.85) | 0.11 (0.42) | 0.23 (0.57) | 0.379 | 0.21 | 0.77 | -0.21 | 0.72 | -0.42 | 0.11 | -0.028 | 1 | -0.24 | 0.56 | 0.18 | 0.67 |
| Actinomyces odontolyticus | 0.19 (0.47) | 0.47 (0.74) | 0.12 (0.32) | 0.047 (0.25) | 0.381 | 0.14 | 0.8 | -0.061 | 0.97 | -0.2 | 0.43 | -0.15 | 0.72 | -0.29 | 0.13 | -0.091 | 0.86 |
| Streptococcus parasanguinis | 0.4 (0.8) | 0.22 (0.54) | 0.11 (0.43) | 0.089 (0.32) | 0.385 | -0.18 | 0.81 | -0.3 | 0.36 | -0.12 | 0.88 | -0.35 | 0.25 | -0.17 | 0.76 | -0.041 | 0.99 |
| Enterococcus faecalis_faecium_hirae | 0.9 (0.99) | 0.77 (0.94) | 0.17 (0.45) | 0.64 (0.83) | 0.393 | -0.19 | 0.91 | -0.65 | 0.08 | -0.46 | 0.25 | -0.2 | 0.87 | -0.012 | 1 | 0.45 | 0.18 |
| Klebsiella aerogenes_pneumoniae | 1.3 (1.3) | 1.7 (1.2) | 1.9 (1.2) | 2 (1.2) | 0.432 | 0.11 | 0.99 | 0.44 | 0.66 | 0.33 | 0.78 | 0.54 | 0.49 | 0.43 | 0.61 | 0.097 | 0.99 |
| Sutterella wadsworthensis | 0 (0) | 0.51 (1.3) | 0.85 (1.5) | 0.52 (1.1) | 0.432 | 0.6 | 0.49 | 0.98 | 0.067 | 0.38 | 0.72 | 0.52 | 0.55 | -0.086 | 0.99 | -0.46 | 0.46 |
| Bacteroides stercoris | 0.38 (0.81) | 0.17 (0.44) | 0.47 (0.98) | 0.44 (0.97) | 0.445 | -0.21 | 0.88 | 0.13 | 0.96 | 0.34 | 0.49 | 0.027 | 1 | 0.24 | 0.75 | -0.1 | 0.96 |
| Blautia luti_massiliensis | 0 (0) | 0.21 (0.67) | 0.58 (0.87) | 0.41 (0.88) | 0.449 | 0.25 | 0.79 | 0.56 | 0.11 | 0.32 | 0.5 | 0.41 | 0.34 | 0.16 | 0.88 | -0.15 | 0.87 |
| Clostridium sartagoforme_tertium | 0.18 (0.47) | 0.25 (0.53) | 0.16 (0.4) | 0.038 (0.2) | 0.453 | -0.077 | 0.92 | -0.063 | 0.94 | 0.014 | 1 | -0.16 | 0.49 | -0.084 | 0.85 | -0.098 | 0.71 |
| Clostridium paraputrificum | 0.32 (0.64) | 0.38 (0.72) | 0.55 (0.65) | 0.71 (0.92) | 0.507 | 0.11 | 0.98 | 0.21 | 0.85 | 0.1 | 0.97 | 0.37 | 0.5 | 0.26 | 0.7 | 0.16 | 0.88 |
| Prevotella copri | 2 (0.76) | 1.8 (0.27) | 2.1 (0.79) | 1.9 (0.66) | 0.52 | -0.2 | 0.84 | 0.14 | 0.92 | 0.34 | 0.34 | -0.13 | 0.93 | 0.065 | 0.99 | -0.27 | 0.42 |
| Fusicatenibacter saccharivorans | 0.076 (0.28) | 0.32 (0.83) | 0.29 (0.77) | 0.25 (0.62) | 0.522 | 0.29 | 0.69 | 0.26 | 0.72 | -0.034 | 1 | 0.17 | 0.9 | -0.12 | 0.95 | -0.088 | 0.97 |
| Streptococcus gallolyticus | 0.34 (0.9) | 0.13 (0.56) | 0.38 (0.84) | 0.18 (0.66) | 0.533 | -0.22 | 0.88 | 0.08 | 0.99 | 0.3 | 0.62 | -0.18 | 0.9 | 0.034 | 1 | -0.26 | 0.61 |
| Alistipes onderdonkii | 0 (0) | 0.15 (0.47) | 0.22 (0.69) | 0.56 (1.1) | 0.548 | 0.18 | 0.88 | 0.25 | 0.67 | 0.074 | 0.98 | 0.56 | 0.061 | 0.38 | 0.24 | 0.31 | 0.32 |
| Bacteroides xylanisolvens | 0.26 (0.67) | 0.68 (1.1) | 0.67 (1.1) | 0.7 (1.2) | 0.577 | 0.46 | 0.59 | 0.53 | 0.4 | 0.068 | 1 | 0.54 | 0.38 | 0.074 | 1 | 0.0054 | 1 |
| Bacteroides galacturonicus_Lactobacillus rogosae | 0 (0) | 0.25 (0.65) | 0.082 (0.31) | 0.23 (0.68) | 0.584 | 0.3 | 0.48 | 0.095 | 0.96 | -0.2 | 0.65 | 0.23 | 0.63 | -0.071 | 0.98 | 0.13 | 0.83 |
| Parabacteroides merdae | 0.62 (0.58) | 0.83 (0.81) | 1.1 (1.2) | 1.2 (1.2) | 0.595 | 0.17 | 0.95 | 0.41 | 0.51 | 0.24 | 0.81 | 0.54 | 0.26 | 0.37 | 0.52 | 0.13 | 0.95 |
| Veillonella ratti_seminalis | 0.52 (1.3) | 0.87 (1.6) | 0.69 (1.2) | 0.88 (1.3) | 0.607 | 0.27 | 0.94 | 0.23 | 0.95 | -0.037 | 1 | 0.32 | 0.88 | 0.05 | 1 | 0.086 | 0.99 |
| Anaerostipes hadrus | 0.066 (0.25) | 0.15 (0.47) | 0.29 (0.67) | 0.32 (0.74) | 0.671 | 0.11 | 0.96 | 0.27 | 0.55 | 0.16 | 0.83 | 0.25 | 0.59 | 0.14 | 0.86 | -0.015 | 1 |
| Enterococcus casseliflavus | 0.37 (0.77) | 0.24 (0.6) | 0.16 (0.48) | 0.23 (0.58) | 0.703 | -0.22 | 0.76 | -0.22 | 0.7 | -0.0033 | 1 | -0.17 | 0.84 | 0.05 | 0.99 | 0.053 | 0.99 |
| Streptococcus equinus_lutetiensis | 0.41 (1.1) | 0.12 (0.52) | 0.39 (0.89) | 0.13 (0.5) | 0.717 | -0.31 | 0.55 | -0.3 | 0.51 | 0.011 | 1 | -0.31 | 0.47 | -0.0012 | 1 | -0.012 | 1 |
| Veillonella parvula | 1.3 (1.1) | 1.2 (1.4) | 1.1 (1.2) | 0.89 (1.2) | 0.719 | -0.11 | 0.99 | -0.065 | 1 | 0.047 | 1 | -0.32 | 0.86 | -0.2 | 0.94 | -0.25 | 0.87 |
| Lachnospira pectinoschiza | 0.19 (0.72) | 0.18 (0.6) | 0.28 (0.79) | 0.51 (1) | 0.757 | -0.0021 | 1 | 0.018 | 1 | 0.02 | 1 | 0.3 | 0.72 | 0.3 | 0.65 | 0.28 | 0.61 |
| Haemophilus parainfluenzae | 1.4 (0.99) | 0.84 (0.85) | 1 (1.1) | 0.92 (0.77) | 0.783 | -0.45 | 0.58 | -0.3 | 0.79 | 0.15 | 0.96 | -0.44 | 0.53 | 0.015 | 1 | -0.13 | 0.96 |
| Streptococcus australis_oralis_peroris_sanguinis | 0.37 (0.75) | 0.65 (0.84) | 0.43 (0.76) | 0.28 (0.57) | 0.794 | 0.095 | 0.98 | -0.11 | 0.96 | -0.2 | 0.76 | -0.12 | 0.95 | -0.21 | 0.72 | -0.011 | 1 |
| Enterococcus avium | 0.35 (0.7) | 0.11 (0.51) | 0.25 (0.59) | 0.29 (0.64) | 0.823 | -0.24 | 0.7 | -0.16 | 0.86 | 0.08 | 0.97 | -0.084 | 0.98 | 0.16 | 0.83 | 0.079 | 0.96 |
| Bacteroides fragilis | 1.1 (1.6) | 1.1 (1.7) | 1.1 (1.5) | 0.77 (1.3) | 0.852 | -0.3 | 0.93 | 0.12 | 0.99 | 0.42 | 0.76 | -0.4 | 0.82 | -0.1 | 1 | -0.52 | 0.51 |
| Clostridium neonatale | 0.48 (1) | 0.73 (1.2) | 0.94 (1.1) | 0.68 (0.95) | 0.858 | 0.33 | 0.85 | 0.37 | 0.76 | 0.043 | 1 | 0.16 | 0.97 | -0.17 | 0.96 | -0.22 | 0.89 |
| Lactobacillus gasseri | 0.64 (0.68) | 0.5 (0.71) | 0.45 (0.65) | 0.47 (0.63) | 0.892 | -0.099 | 0.98 | -0.28 | 0.63 | -0.18 | 0.83 | -0.22 | 0.77 | -0.12 | 0.94 | 0.058 | 0.99 |
| Faecalibacterium prausnitzii | 0.85 (0.86) | 1.2 (0.9) | 1.2 (1.1) | 1.2 (0.97) | 0.919 | 0.37 | 0.73 | 0.31 | 0.77 | -0.053 | 1 | 0.24 | 0.88 | -0.12 | 0.98 | -0.071 | 0.99 |
| Eggerthella lenta | 0.49 (0.7) | 0.54 (0.62) | 0.3 (0.54) | 0.49 (0.6) | 0.945 | -0.045 | 1 | -0.18 | 0.84 | -0.13 | 0.9 | -0.041 | 1 | 0.0032 | 1 | 0.14 | 0.86 |
| Campylobacter concisus | 0.16 (0.45) | 0.087 (0.27) | 0.28 (0.6) | 0.15 (0.43) | 0.947 | -0.067 | 0.97 | -0.028 | 1 | 0.039 | 0.99 | -0.024 | 1 | 0.043 | 0.98 | 0.0044 | 1 |
| Helicobacter felis | 0.3 (0.6) | 0.21 (0.52) | 0.32 (0.59) | 0.29 (0.55) | 0.95 | -0.08 | 0.98 | -0.059 | 0.99 | 0.022 | 1 | -0.04 | 1 | 0.041 | 1 | 0.019 | 1 |
| Staphylococcus aureus_capitis_epidermidis_haemolyticus_hominis_pasteuri_petrasii_warneri | 0.28 (0.57) | 0.24 (0.53) | 0.33 (0.58) | 0.41 (0.65) | 0.978 | -0.012 | 1 | 0.018 | 1 | 0.03 | 1 | 0.11 | 0.95 | 0.12 | 0.92 | 0.089 | 0.95 |

**Supplemental Table 6**: Metabolic phenotype association with milk consumption type.

|  |  |  |  |  |  | ***BP-BB*** | | ***TF-BB*** | | ***TF-BP*** | | ***ASF-BB*** | | ***ASF-BP*** | | ***ASF-TF*** | |
| --- | --- | --- | --- | --- | --- | --- | --- | --- | --- | --- | --- | --- | --- | --- | --- | --- | --- |
| **Tax** | **BB (n=14) Mean (SD)** | **BP (n=20) Mean (SD)** | **TF (n=30) Mean (SD)** | **ASF (n=27) Mean (SD)** | **ANOVA *p*-value** | **Mean Difference** | ***p*-value** | **Mean Difference** | ***p*-value** | **Mean Difference** | ***p*-value** | **Mean Difference** | ***p*-value** | **Mean Difference** | ***p*-value** | **Mean Difference** | ***p*-value** |
| **Carbohydrate Uptake and Formation** | | |  |  |  |  |  |  |  |  |  |  |  |  |  |  |  |
| Gtl | 0.26 (0.15) | 0.15 (0.17) | 0.085 (0.12) | 0.056 (0.044) | 3.41E-06 | -0.13 | 0.0022 | -0.19 | 1.00E-06 | -0.057 | 0.23 | -0.2 | 1.70E-07 | -0.071 | 0.09 | -0.013 | 0.96 |
| (GlcA)n | 0.25 (0.17) | 0.14 (0.17) | 0.069 (0.11) | 0.039 (0.03) | 4.65E-06 | -0.12 | 0.0033 | -0.19 | 2.50E-07 | -0.071 | 0.077 | -0.21 | 2.40E-08 | -0.087 | 0.017 | -0.017 | 0.91 |
| Lac | 0.61 (0.27) | 0.5 (0.24) | 0.28 (0.16) | 0.26 (0.18) | 8.67E-06 | -0.14 | 0.26 | -0.37 | 8.50E-06 | -0.23 | 0.0038 | -0.37 | 8.10E-06 | -0.23 | 0.0038 | 0.0015 | 1 |
| GalN | 0.27 (0.16) | 0.17 (0.17) | 0.1 (0.13) | 0.066 (0.065) | 9.93E-05 | -0.12 | 0.023 | -0.18 | 9.70E-05 | -0.056 | 0.4 | -0.21 | 6.00E-06 | -0.084 | 0.096 | -0.027 | 0.82 |
| ddGlcA | 0.3 (0.19) | 0.16 (0.18) | 0.11 (0.14) | 0.094 (0.097) | 0.000107 | -0.16 | 0.0057 | -0.2 | 8.50E-05 | -0.042 | 0.73 | -0.21 | 5.00E-05 | -0.047 | 0.65 | -0.0049 | 1 |
| Mtl | 0.37 (0.17) | 0.26 (0.18) | 0.19 (0.15) | 0.16 (0.1) | 0.000134 | -0.14 | 0.05 | -0.21 | 0.00037 | -0.067 | 0.43 | -0.22 | 0.00014 | -0.079 | 0.28 | -0.012 | 0.99 |
| GalNAc | 0.24 (0.14) | 0.14 (0.15) | 0.089 (0.11) | 0.056 (0.06) | 0.000141 | -0.11 | 0.028 | -0.16 | 0.00012 | -0.051 | 0.39 | -0.18 | 8.50E-06 | -0.074 | 0.099 | -0.023 | 0.84 |
| Psi-Lys | 0.17 (0.12) | 0.11 (0.11) | 0.06 (0.08) | 0.038 (0.03) | 0.000355 | -0.075 | 0.027 | -0.12 | 3.60E-05 | -0.043 | 0.22 | -0.13 | 3.00E-06 | -0.057 | 0.051 | -0.015 | 0.88 |
| a(Xyl)n | 0.31 (0.21) | 0.16 (0.16) | 0.14 (0.18) | 0.086 (0.12) | 0.000437 | -0.17 | 0.019 | -0.17 | 0.01 | 0.0024 | 1 | -0.23 | 0.00025 | -0.056 | 0.65 | -0.059 | 0.52 |
| Gnt | 0.37 (0.2) | 0.29 (0.21) | 0.18 (0.16) | 0.15 (0.13) | 0.00159 | -0.094 | 0.37 | -0.21 | 0.00098 | -0.12 | 0.085 | -0.23 | 0.00039 | -0.13 | 0.043 | -0.013 | 0.99 |
| Tre | 0.33 (0.2) | 0.19 (0.2) | 0.16 (0.2) | 0.12 (0.11) | 0.00239 | -0.16 | 0.035 | -0.18 | 0.0069 | -0.019 | 0.98 | -0.21 | 0.0013 | -0.046 | 0.79 | -0.028 | 0.92 |
| Fru-Lys | 0.21 (0.12) | 0.13 (0.12) | 0.12 (0.12) | 0.089 (0.06) | 0.003 | -0.096 | 0.046 | -0.11 | 0.011 | -0.01 | 0.99 | -0.13 | 0.0016 | -0.03 | 0.75 | -0.02 | 0.88 |
| Raf | 0.2 (0.13) | 0.23 (0.21) | 0.088 (0.08) | 0.087 (0.11) | 0.00312 | 0.028 | 0.95 | -0.12 | 0.076 | -0.14 | 0.008 | -0.13 | 0.041 | -0.16 | 0.0033 | -0.012 | 0.99 |
| Mel | 0.25 (0.17) | 0.15 (0.14) | 0.12 (0.14) | 0.1 (0.11) | 0.00377 | -0.12 | 0.063 | -0.14 | 0.0082 | -0.024 | 0.93 | -0.15 | 0.005 | -0.03 | 0.87 | -0.0063 | 1 |
| b(Ara)n | 0.14 (0.097) | 0.15 (0.15) | 0.052 (0.057) | 0.05 (0.08) | 0.00806 | 0.003 | 1 | -0.088 | 0.051 | -0.091 | 0.022 | -0.097 | 0.026 | -0.1 | 0.0095 | -0.0084 | 0.99 |
| Srl | 0.31 (0.19) | 0.2 (0.19) | 0.15 (0.15) | 0.18 (0.13) | 0.00998 | -0.13 | 0.056 | -0.17 | 0.0027 | -0.04 | 0.79 | -0.13 | 0.027 | -0.0025 | 1 | 0.038 | 0.75 |
| Ara | 0.69 (0.16) | 0.51 (0.22) | 0.45 (0.22) | 0.5 (0.18) | 0.0159 | -0.16 | 0.12 | -0.21 | 0.013 | -0.047 | 0.87 | -0.18 | 0.048 | -0.014 | 1 | 0.034 | 0.93 |
| Fru | 0.94 (0.032) | 0.87 (0.14) | 0.82 (0.16) | 0.78 (0.15) | 0.0172 | -0.095 | 0.27 | -0.12 | 0.073 | -0.024 | 0.95 | -0.17 | 0.0045 | -0.071 | 0.36 | -0.048 | 0.61 |
| Lnb | 0.28 (0.16) | 0.28 (0.18) | 0.17 (0.13) | 0.17 (0.14) | 0.0259 | -0.015 | 0.99 | -0.14 | 0.054 | -0.12 | 0.065 | -0.13 | 0.057 | -0.12 | 0.069 | 0.0021 | 1 |
| Rha | 0.52 (0.19) | 0.37 (0.22) | 0.43 (0.21) | 0.48 (0.21) | 0.0472 | -0.12 | 0.34 | -0.05 | 0.87 | 0.068 | 0.66 | -0.015 | 1 | 0.1 | 0.3 | 0.035 | 0.91 |
| (Rha)n | 0.27 (0.26) | 0.25 (0.22) | 0.35 (0.22) | 0.43 (0.21) | 0.053 | 0.031 | 0.97 | 0.14 | 0.1 | 0.11 | 0.2 | 0.19 | 0.011 | 0.16 | 0.024 | 0.051 | 0.73 |
| Mal | 0.83 (0.16) | 0.78 (0.17) | 0.76 (0.2) | 0.73 (0.14) | 0.0745 | -0.052 | 0.78 | -0.028 | 0.95 | 0.024 | 0.95 | -0.097 | 0.22 | -0.045 | 0.76 | -0.069 | 0.33 |
| (Man)n | 0.22 (0.15) | 0.33 (0.22) | 0.34 (0.2) | 0.3 (0.16) | 0.0752 | 0.11 | 0.31 | 0.16 | 0.034 | 0.051 | 0.77 | 0.09 | 0.4 | -0.018 | 0.98 | -0.069 | 0.44 |
| Bgl | 0.46 (0.19) | 0.35 (0.19) | 0.38 (0.2) | 0.34 (0.16) | 0.078 | -0.12 | 0.25 | -0.1 | 0.35 | 0.022 | 0.98 | -0.14 | 0.11 | -0.014 | 0.99 | -0.036 | 0.88 |
| GalA | 0.55 (0.2) | 0.38 (0.22) | 0.43 (0.24) | 0.46 (0.23) | 0.085 | -0.14 | 0.3 | -0.086 | 0.61 | 0.049 | 0.87 | -0.059 | 0.83 | 0.076 | 0.64 | 0.027 | 0.96 |
| Bga | 0.22 (0.14) | 0.26 (0.18) | 0.14 (0.11) | 0.16 (0.14) | 0.0941 | 0.045 | 0.85 | -0.082 | 0.37 | -0.13 | 0.039 | -0.07 | 0.5 | -0.12 | 0.069 | 0.011 | 0.99 |
| (Mal)n | 0.8 (0.16) | 0.76 (0.17) | 0.72 (0.19) | 0.7 (0.14) | 0.103 | -0.046 | 0.83 | -0.032 | 0.92 | 0.015 | 0.99 | -0.096 | 0.23 | -0.05 | 0.7 | -0.064 | 0.4 |
| Man | 0.49 (0.21) | 0.37 (0.22) | 0.41 (0.22) | 0.39 (0.2) | 0.12 | -0.1 | 0.49 | -0.061 | 0.78 | 0.039 | 0.92 | -0.084 | 0.56 | 0.016 | 0.99 | -0.023 | 0.97 |
| Glc | 0.89 (0.13) | 0.81 (0.16) | 0.76 (0.17) | 0.77 (0.11) | 0.132 | -0.09 | 0.31 | -0.1 | 0.15 | -0.013 | 0.99 | -0.12 | 0.067 | -0.029 | 0.91 | -0.016 | 0.98 |
| NANa | 0.57 (0.2) | 0.53 (0.21) | 0.58 (0.21) | 0.64 (0.19) | 0.138 | -0.029 | 0.98 | 0.07 | 0.69 | 0.099 | 0.33 | 0.1 | 0.39 | 0.13 | 0.13 | 0.03 | 0.94 |
| Xyl | 0.59 (0.22) | 0.49 (0.23) | 0.55 (0.21) | 0.57 (0.19) | 0.165 | -0.096 | 0.55 | 0.021 | 0.99 | 0.12 | 0.23 | 0.0064 | 1 | 0.1 | 0.34 | -0.015 | 0.99 |
| Chb | 0.77 (0.22) | 0.68 (0.21) | 0.56 (0.23) | 0.58 (0.2) | 0.19 | -0.097 | 0.58 | -0.17 | 0.077 | -0.075 | 0.65 | -0.17 | 0.072 | -0.077 | 0.63 | -0.0011 | 1 |
| Ino | 0.095 (0.17) | 0.083 (0.084) | 0.12 (0.12) | 0.17 (0.13) | 0.199 | -0.024 | 0.95 | 0.014 | 0.98 | 0.038 | 0.74 | 0.065 | 0.38 | 0.089 | 0.089 | 0.051 | 0.42 |
| Rtl | 0.063 (0.17) | 0.023 (0.026) | 0.044 (0.11) | 0.041 (0.057) | 0.214 | -0.051 | 0.49 | -0.025 | 0.87 | 0.026 | 0.83 | -0.026 | 0.86 | 0.025 | 0.84 | -0.00071 | 1 |
| GlcA | 0.61 (0.19) | 0.52 (0.23) | 0.58 (0.21) | 0.56 (0.2) | 0.218 | -0.086 | 0.63 | 0.035 | 0.95 | 0.12 | 0.2 | -0.022 | 0.99 | 0.064 | 0.71 | -0.057 | 0.71 |
| Atl | 0.065 (0.18) | 0.024 (0.026) | 0.045 (0.11) | 0.041 (0.061) | 0.219 | -0.053 | 0.49 | -0.026 | 0.87 | 0.026 | 0.84 | -0.029 | 0.84 | 0.024 | 0.87 | -0.0021 | 1 |
| Scr | 0.77 (0.15) | 0.72 (0.19) | 0.68 (0.19) | 0.66 (0.15) | 0.24 | -0.064 | 0.72 | -0.054 | 0.77 | 0.0097 | 1 | -0.11 | 0.21 | -0.045 | 0.81 | -0.055 | 0.61 |
| FOS | 0.35 (0.29) | 0.41 (0.24) | 0.48 (0.25) | 0.51 (0.19) | 0.249 | 0.088 | 0.64 | 0.2 | 0.022 | 0.12 | 0.27 | 0.19 | 0.043 | 0.097 | 0.42 | -0.019 | 0.99 |
| Rib | 0.42 (0.2) | 0.34 (0.24) | 0.29 (0.21) | 0.31 (0.2) | 0.268 | -0.11 | 0.43 | -0.15 | 0.11 | -0.042 | 0.9 | -0.12 | 0.25 | -0.013 | 1 | 0.029 | 0.95 |
| Fuc | 0.46 (0.2) | 0.43 (0.22) | 0.32 (0.21) | 0.33 (0.17) | 0.283 | -0.084 | 0.64 | -0.14 | 0.15 | -0.055 | 0.79 | -0.14 | 0.16 | -0.053 | 0.81 | 0.0019 | 1 |
| b(Xyl)n | 0.47 (0.24) | 0.52 (0.23) | 0.57 (0.22) | 0.6 (0.16) | 0.302 | 0.065 | 0.75 | 0.17 | 0.034 | 0.1 | 0.25 | 0.14 | 0.09 | 0.078 | 0.49 | -0.025 | 0.96 |
| (Fuc)n | 0.13 (0.14) | 0.23 (0.21) | 0.19 (0.2) | 0.22 (0.17) | 0.302 | 0.077 | 0.66 | 0.079 | 0.58 | 0.002 | 1 | 0.09 | 0.47 | 0.012 | 1 | 0.011 | 1 |
| Gal | 0.65 (0.17) | 0.58 (0.2) | 0.65 (0.2) | 0.66 (0.19) | 0.319 | -0.06 | 0.83 | 0.057 | 0.81 | 0.12 | 0.21 | 0.031 | 0.96 | 0.091 | 0.41 | -0.026 | 0.96 |
| Fru-Asp | 5e-05 (0.00017) | 0.003 (0.0099) | 0.0021 (0.0047) | 0.0043 (0.0099) | 0.359 | 0.0031 | 0.71 | 0.0021 | 0.85 | -0.00093 | 0.98 | 0.0043 | 0.37 | 0.0012 | 0.96 | 0.0021 | 0.76 |
| Tag | 0.026 (0.068) | 0.015 (0.021) | 0.029 (0.065) | 0.022 (0.028) | 0.373 | -0.018 | 0.75 | 0.00069 | 1 | 0.019 | 0.62 | -0.0054 | 0.99 | 0.013 | 0.84 | -0.0061 | 0.97 |
| GlcNAc | 0.67 (0.24) | 0.59 (0.2) | 0.61 (0.22) | 0.61 (0.18) | 0.39 | -0.083 | 0.66 | -0.03 | 0.97 | 0.053 | 0.82 | -0.034 | 0.96 | 0.049 | 0.85 | -0.004 | 1 |
| (GalA)n | 0.32 (0.28) | 0.24 (0.22) | 0.34 (0.24) | 0.37 (0.2) | 0.393 | -0.042 | 0.95 | 0.066 | 0.79 | 0.11 | 0.36 | 0.077 | 0.71 | 0.12 | 0.27 | 0.011 | 1 |
| Hyl | 0.026 (0.051) | 0.037 (0.054) | 0.051 (0.071) | 0.047 (0.045) | 0.405 | 0.0083 | 0.98 | 0.027 | 0.55 | 0.018 | 0.75 | 0.02 | 0.74 | 0.012 | 0.91 | -0.0063 | 0.98 |
| ManNAc | 0.0086 (0.0078) | 0.0075 (0.011) | 0.016 (0.029) | 0.0083 (0.0071) | 0.422 | -9.00E-04 | 1 | 0.0054 | 0.78 | 0.0063 | 0.62 | -0.00085 | 1 | 4.70E-05 | 1 | -0.0062 | 0.52 |
| Xtl | 0.049 (0.046) | 0.082 (0.15) | 0.047 (0.072) | 0.041 (0.037) | 0.422 | 0.031 | 0.8 | -0.0036 | 1 | -0.034 | 0.63 | -0.011 | 0.98 | -0.042 | 0.46 | -0.0074 | 0.99 |
| Glc-Lys | 0.046 (0.091) | 0.033 (0.038) | 0.058 (0.089) | 0.049 (0.049) | 0.433 | -0.023 | 0.81 | 0.0079 | 0.99 | 0.031 | 0.49 | -0.00027 | 1 | 0.023 | 0.72 | -0.0082 | 0.97 |
| Aga | 0.066 (0.059) | 0.087 (0.094) | 0.089 (0.093) | 0.11 (0.098) | 0.713 | 0.023 | 0.91 | 0.022 | 0.91 | -0.0015 | 1 | 0.037 | 0.67 | 0.013 | 0.97 | 0.015 | 0.94 |
| a(Ara)n | 0.46 (0.24) | 0.41 (0.22) | 0.41 (0.24) | 0.46 (0.18) | 0.874 | -0.0074 | 1 | 0.0045 | 1 | 0.012 | 1 | 0.022 | 0.99 | 0.029 | 0.96 | 0.017 | 0.99 |
| **Vitamin Biosynthesis** |  |  |  |  |  |  |  |  |  |  |  |  |  |  |  |  |  |
| B12 | 0.3 (0.21) | 0.45 (0.23) | 0.53 (0.21) | 0.65 (0.17) | 4.91E-05 | 0.17 | 0.076 | 0.29 | 0.00024 | 0.11 | 0.25 | 0.38 | 9.60E-07 | 0.2 | 0.0056 | 0.092 | 0.31 |
| B2 | 0.71 (0.14) | 0.67 (0.21) | 0.8 (0.12) | 0.8 (0.16) | 0.0244 | -0.038 | 0.92 | 0.11 | 0.24 | 0.15 | 0.032 | 0.11 | 0.24 | 0.14 | 0.032 | -0.0014 | 1 |
| B1 | 0.89 (0.091) | 0.81 (0.17) | 0.71 (0.21) | 0.74 (0.14) | 0.0483 | -0.084 | 0.51 | -0.15 | 0.047 | -0.064 | 0.6 | -0.14 | 0.062 | -0.056 | 0.68 | 0.0073 | 1 |
| B3 | 0.87 (0.14) | 0.77 (0.18) | 0.75 (0.17) | 0.77 (0.12) | 0.101 | -0.11 | 0.13 | -0.083 | 0.31 | 0.032 | 0.89 | -0.092 | 0.22 | 0.023 | 0.95 | -0.0089 | 1 |
| B6 | 0.88 (0.14) | 0.81 (0.16) | 0.78 (0.12) | 0.77 (0.15) | 0.112 | -0.081 | 0.38 | -0.081 | 0.31 | 0.00062 | 1 | -0.11 | 0.094 | -0.028 | 0.91 | -0.028 | 0.87 |
| B5 | 0.62 (0.18) | 0.55 (0.22) | 0.64 (0.18) | 0.64 (0.21) | 0.132 | -0.068 | 0.78 | 0.071 | 0.71 | 0.14 | 0.11 | 0.048 | 0.89 | 0.12 | 0.23 | -0.023 | 0.97 |
| B9 | 0.96 (0.054) | 0.9 (0.17) | 0.9 (0.12) | 0.86 (0.11) | 0.185 | -0.069 | 0.46 | -0.072 | 0.35 | -0.0031 | 1 | -0.099 | 0.11 | -0.03 | 0.87 | -0.027 | 0.87 |
| B7 | 0.6 (0.22) | 0.55 (0.23) | 0.57 (0.23) | 0.64 (0.23) | 0.191 | -0.047 | 0.94 | 0.0027 | 1 | 0.049 | 0.88 | 0.07 | 0.77 | 0.12 | 0.31 | 0.067 | 0.67 |
| Lipoate | 0.52 (0.25) | 0.45 (0.24) | 0.5 (0.24) | 0.54 (0.25) | 0.195 | -0.06 | 0.89 | 0.026 | 0.99 | 0.086 | 0.6 | 0.056 | 0.88 | 0.12 | 0.34 | 0.03 | 0.96 |
| Q | 0.61 (0.22) | 0.58 (0.22) | 0.63 (0.19) | 0.66 (0.22) | 0.22 | -0.025 | 0.99 | 0.05 | 0.89 | 0.076 | 0.64 | 0.077 | 0.69 | 0.1 | 0.39 | 0.026 | 0.97 |
| K | 0.65 (0.16) | 0.55 (0.23) | 0.62 (0.2) | 0.59 (0.18) | 0.254 | -0.1 | 0.49 | 0.0019 | 1 | 0.11 | 0.32 | -0.039 | 0.94 | 0.066 | 0.71 | -0.041 | 0.88 |
| **Short Chain Fatty Acid Produ ction** |  |  |  |  |  |  |  |  |  |  |  |  |  |  |  |  |  |
| Butyrate | 0.01 (0.013) | 0.075 (0.14) | 0.1 (0.12) | 0.11 (0.1) | 0.122 | 0.075 | 0.31 | 0.09 | 0.12 | 0.015 | 0.98 | 0.1 | 0.056 | 0.027 | 0.88 | 0.013 | 0.98 |
| Propionate | 0.37 (0.22) | 0.5 (0.24) | 0.58 (0.23) | 0.7 (0.19) | 5.86E-05 | 0.15 | 0.18 | 0.27 | 0.00066 | 0.12 | 0.19 | 0.36 | 3.80E-06 | 0.21 | 0.0041 | 0.089 | 0.34 |
| **Amino Acid Biosynthesis** |  |  |  |  |  |  |  |  |  |  |  |  |  |  |  |  |  |
| Cys | 0.65 (0.19) | 0.61 (0.25) | 0.79 (0.15) | 0.8 (0.15) | 0.00205 | -0.033 | 0.96 | 0.18 | 0.023 | 0.22 | 0.0019 | 0.17 | 0.031 | 0.21 | 0.0027 | -0.0078 | 1 |
| Thr | 0.99 (0.014) | 0.99 (0.021) | 0.97 (0.072) | 0.93 (0.14) | 0.00604 | -0.0054 | 1 | -0.02 | 0.8 | -0.014 | 0.89 | -0.068 | 0.011 | -0.062 | 0.011 | -0.048 | 0.035 |
| Trp | 0.98 (0.02) | 0.93 (0.13) | 0.89 (0.076) | 0.87 (0.09) | 0.0263 | -0.057 | 0.3 | -0.076 | 0.061 | -0.019 | 0.9 | -0.1 | 0.0054 | -0.045 | 0.36 | -0.026 | 0.71 |
| Met | 0.99 (0.0085) | 0.99 (0.016) | 0.98 (0.05) | 0.97 (0.033) | 0.0433 | -0.0071 | 0.84 | -0.0057 | 0.89 | 0.0014 | 1 | -0.02 | 0.069 | -0.012 | 0.32 | -0.014 | 0.14 |
| Tyr | 0.99 (0.0057) | 0.98 (0.025) | 0.97 (0.036) | 0.97 (0.032) | 0.066 | -0.017 | 0.42 | -0.026 | 0.064 | -0.0087 | 0.79 | -0.025 | 0.071 | -0.0081 | 0.82 | 0.00062 | 1 |
| Asn | 0.65 (0.21) | 0.59 (0.25) | 0.72 (0.2) | 0.72 (0.17) | 0.13 | -0.049 | 0.91 | 0.13 | 0.24 | 0.17 | 0.028 | 0.093 | 0.5 | 0.14 | 0.1 | -0.033 | 0.93 |
| Pro | 1 (0.0043) | 0.99 (0.01) | 1 (0.005) | 0.98 (0.032) | 0.214 | -0.0032 | 0.97 | 0.00035 | 1 | 0.0036 | 0.93 | -0.011 | 0.3 | -0.0082 | 0.52 | -0.012 | 0.13 |
| Chor | 1 (0.0011) | 1 (0.0067) | 1 (0.0012) | 1 (0.0052) | 0.259 | -0.0029 | 0.31 | 0.00059 | 0.98 | 0.0035 | 0.074 | -0.00074 | 0.96 | 0.0022 | 0.42 | -0.0013 | 0.71 |
| Lys | 1 (0.0023) | 0.99 (0.016) | 0.98 (0.052) | 0.98 (0.028) | 0.312 | -0.0047 | 0.95 | -0.0069 | 0.82 | -0.0022 | 0.99 | -0.015 | 0.22 | -0.011 | 0.46 | -0.0085 | 0.55 |
| Ile_Val | 0.99 (0.0089) | 0.96 (0.052) | 0.92 (0.11) | 0.96 (0.045) | 0.386 | -0.032 | 0.49 | -0.048 | 0.099 | -0.016 | 0.82 | -0.033 | 0.37 | -0.0014 | 1 | 0.015 | 0.81 |
| Leu | 0.99 (0.0093) | 0.96 (0.053) | 0.92 (0.11) | 0.95 (0.045) | 0.44 | -0.03 | 0.52 | -0.046 | 0.12 | -0.015 | 0.85 | -0.031 | 0.44 | -0.00034 | 1 | 0.015 | 0.8 |
| Asp | 1 (0.00085) | 1 (0.0028) | 1 (0.0039) | 0.99 (0.027) | 0.579 | -0.0011 | 1 | -0.00087 | 1 | 0.00023 | 1 | -0.0064 | 0.63 | -0.0053 | 0.7 | -0.0055 | 0.59 |
| Gln | 1 (0.0015) | 1 (0.0051) | 1 (0.0043) | 0.99 (0.028) | 0.633 | -0.0017 | 0.99 | -0.001 | 1 | 0.00069 | 1 | -0.0064 | 0.66 | -0.0047 | 0.79 | -0.0054 | 0.63 |
| Ser | 0.93 (0.11) | 0.89 (0.13) | 0.9 (0.11) | 0.92 (0.062) | 0.644 | -0.037 | 0.71 | -0.0079 | 0.99 | 0.029 | 0.75 | -0.006 | 1 | 0.031 | 0.71 | 0.0019 | 1 |
| Gly | 0.93 (0.11) | 0.91 (0.14) | 0.93 (0.071) | 0.94 (0.066) | 0.707 | -0.026 | 0.88 | 0.0077 | 1 | 0.033 | 0.67 | 0.008 | 0.99 | 0.034 | 0.65 | 0.00031 | 1 |
| His | 0.98 (0.015) | 0.98 (0.048) | 0.96 (0.059) | 0.96 (0.042) | 0.721 | -0.0098 | 0.9 | -0.012 | 0.81 | -0.0018 | 1 | -0.02 | 0.43 | -0.01 | 0.83 | -0.0083 | 0.86 |
| Arg | 0.98 (0.019) | 0.98 (0.023) | 0.96 (0.069) | 0.97 (0.034) | 0.772 | -0.0022 | 1 | 0.00025 | 1 | 0.0025 | 0.99 | -0.0073 | 0.86 | -0.0051 | 0.93 | -0.0076 | 0.75 |
| Phe | 0.99 (0.0062) | 0.99 (0.013) | 0.99 (0.023) | 0.99 (0.033) | 0.797 | -0.0053 | 0.94 | -0.0013 | 1 | 0.004 | 0.95 | -0.0074 | 0.81 | -0.0021 | 0.99 | -0.006 | 0.81 |
| Glu | 1 (0.00086) | 1 (0.0028) | 1 (0.0039) | 1 (0.005) | 0.904 | -0.0011 | 0.88 | -0.00089 | 0.92 | 0.00021 | 1 | -0.0011 | 0.85 | -3.70E-06 | 1 | -0.00022 | 1 |
| **Amino Acid Degradation** |  |  |  |  |  |  |  |  |  |  |  |  |  |  |  |  |  |
| Lys_D | 0.3 (0.19) | 0.18 (0.18) | 0.13 (0.15) | 0.13 (0.11) | 0.00188 | -0.15 | 0.018 | -0.19 | 0.00055 | -0.039 | 0.78 | -0.17 | 0.0013 | -0.026 | 0.92 | 0.013 | 0.98 |
| Met_D | 0.3 (0.18) | 0.29 (0.24) | 0.12 (0.11) | 0.12 (0.14) | 0.00231 | -0.017 | 0.99 | -0.2 | 0.0043 | -0.18 | 0.0043 | -0.19 | 0.0059 | -0.18 | 0.006 | 0.007 | 1 |
| Trp_D | 0.39 (0.18) | 0.27 (0.19) | 0.25 (0.18) | 0.28 (0.16) | 0.0855 | -0.14 | 0.12 | -0.14 | 0.057 | -0.0077 | 1 | -0.11 | 0.18 | 0.023 | 0.97 | 0.031 | 0.9 |
| His_D | 0.3 (0.27) | 0.36 (0.26) | 0.41 (0.25) | 0.46 (0.22) | 0.11 | 0.072 | 0.81 | 0.17 | 0.095 | 0.1 | 0.44 | 0.19 | 0.066 | 0.11 | 0.34 | 0.011 | 1 |
| Pro_D | 0.46 (0.26) | 0.35 (0.22) | 0.35 (0.23) | 0.39 (0.24) | 0.183 | -0.12 | 0.42 | -0.071 | 0.75 | 0.047 | 0.89 | -0.043 | 0.93 | 0.075 | 0.65 | 0.028 | 0.96 |
| Thr_D | 0.51 (0.24) | 0.45 (0.23) | 0.49 (0.22) | 0.54 (0.25) | 0.184 | -0.052 | 0.91 | 0.036 | 0.96 | 0.088 | 0.56 | 0.06 | 0.84 | 0.11 | 0.34 | 0.025 | 0.98 |
| Leu_D | 0 (0) | 8.8e-06 (3.9e-05) | 0.00037 (0.0013) | 0.0032 (0.011) | 0.348 | -7.00E-18 | 1 | 0.00031 | 1 | 0.00031 | 1 | 0.0032 | 0.33 | 0.0032 | 0.26 | 0.0029 | 0.25 |
| Val_D | 1e-05 (3.8e-05) | 2.9e-06 (1.3e-05) | 0.00012 (0.00039) | 0.0016 (0.0073) | 0.982 | -7.50E-06 | 1 | 0.00011 | 1 | 0.00012 | 1 | 0.0016 | 0.29 | 0.0016 | 0.22 | 0.0015 | 0.19 |
